# Early life conditions influence fledging success and subsequent local recruitment rates in a declining migratory songbird, the Whinchat *Saxicola rubetra*

**DOI:** 10.1002/ece3.10346

**Published:** 2023-07-20

**Authors:** Chay Halliwell, Martin Ketcher, Amanda Proud, Stephen Westerberg, David J. T. Douglas, Malcolm D. Burgess

**Affiliations:** ^1^ School of Biosciences University of Sheffield Sheffield UK; ^2^ RSPB Geltsdale Brampton UK; ^3^ RSPB Centre for Conservation Science, RSPB Scotland Edinburgh UK; ^4^ RSPB Centre for Conservation Science, The Lodge Sandy UK

**Keywords:** Afro‐Palearctic, brood size, migration, post‐fledging survival, rainfall, silver spoon effect

## Abstract

Life history traits and environmental conditions influence reproductive success in animals, and consequences of these can influence subsequent survival and recruitment into breeding populations. Understanding influences on demographic rates is required to determine the causes of decline. Migratory species experience spatially and temporally variable conditions across their annual cycle, making identifying where the factors influencing demographic rates operate challenging. Here, we use the Whinchat *Saxicola rubetra* as a model declining long‐distance migrant bird. We analyse 10 years of data from 247 nesting attempts and 2519 post‐fledging observations of 1193 uniquely marked nestlings to examine the influence of life history traits, habitat characteristics and weather on survival of young from the nestling stage to local recruitment into the natal population. We detected potential silver spoon effects where conditions during the breeding stage influence subsequent apparent local recruitment rates, with higher recruitment for fledglings from larger broods, and recruitment rate negatively related to rainfall that chicks experienced in‐nest. Additionally, extreme temperatures experienced pre‐ and post‐fledging increased fledging success and recruitment rate. However, we could not determine whether this was driven by temperature influencing mortality during the post‐fledging period or later in the annual cycle. Brood size declined with hatching date. In‐nest survival increased with brood size and was highest at local temperature extremes. Furthermore, nest survival was highest at nests surrounded with 40%–60% vegetation cover of Bracken *Pteridium aquilinum* within 50 m of the nest. Our results show that breeding phenology and environmental factors may influence fledging success and recruitment in songbird populations, with conditions experienced during the nestling stage influencing local recruitment rates in Whinchats (i.e. silver spoon effect). Recruitment rates are key drivers of songbird population dynamics. Our results help identify some of the likely breeding season mechanisms that could be important population drivers.

## Graphical abstract

Conditions that young birds experience pre‐ and post‐fledging may influence their immediate survival changes and subsequent survival later in life. We found that offspring from larger broods were more likely to fledge and to recruit into the local breeding population, while nests with intermediate bracken cover were more likely to successfully fledge. Rainfall experienced by chicks in‐nest appears to influence survival in subsequent stages of their life cycle, though we could not determine whether this effect occurred during the critical post‐fledging period or later in their life.

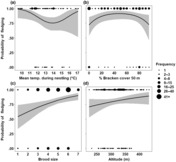

## 1 INTRODUCTION

Across Europe many migratory bird species have undergone substantial population declines over recent decades, with long‐distance Afro‐Palearctic migrants experiencing particularly severe declines (Vickery et al., 2014). Although the causes remain largely undiagnosed, declines could be driven by changes in productivity or impacts on survival at one or more locations across their annual cycle, that is, at breeding sites, non‐breeding sites or during migration (Howard et al., 2020; Vickery et al., 2014). Furthermore, conditions individuals experience during one stage can affect productivity and survival rates in subsequent stages, that is, carry‐over effects (Harrison et al., 2011). Carry‐over effects are typically considered non‐lethal effects; however, they can apply to scenarios where conditions experienced during one stage influence survival rates in subsequent seasons (Harrison et al., 2011). By this definition, if conditions experienced during early life influenced survival in subsequent seasons of the annual cycle this effect would be considered both a silver spoon (Cockburn, 1991; Grafen, 1988) and carry‐over effect. Carry‐over effects have been detected in many migratory birds, particularly effects linking non‐breeding (Rushing et al., 2016) and migratory conditions (Finch et al., 2014) to reproductive stages. However, there is increasing evidence that breeding site conditions may influence migrants' survival and condition in subsequent stages of the annual cycle (Akresh et al., 2021; Evans et al., 2020; Latta et al., 2016), whether in the critical post‐fledging period (e.g. Evans et al., 2020; Naef‐Daenzer & Grüebler, 2016) or during the non‐breeding season (e.g. Latta et al., 2016).

After hatching, many bird species experience high pre‐fledging mortality, and although less studied, the period between leaving the nest (fledging) and independence from parental care is also often a period with high mortality (Cox et al., 2014; Naef‐Daenzer & Grüebler, 2016). For declining migratory species, disentangling post‐fledging mortality from first year mortality is difficult but can help identify whether pressures occur in breeding or non‐breeding areas, which is useful knowledge for guiding conservation effort. Migratory birds face potential impacts on survival at multiple stages and locations across their annual cycle, with each stage and location a potential survival bottleneck (Faaborg et al., 2010). Therefore, migrants are more likely to experience environmental change which could impact survival (‘Multiple jeopardy’; Newton, 2004).

In this study, we combine 10 years of detailed breeding data from 247 nests and resighting data from 1193 individually marked nestling Whinchats *Saxicola rubetra* to examine the influence of environmental drivers on stage specific survival and local recruitment. Like many other Afro‐Palearctic migrants, Whinchat populations have decreased substantially over recent decades, 89% since 1980 across Europe (PECBMS, 2020) and 57% since 1995 in the UK (Woodward et al., 2020). Previous studies indicate that breeding productivity and dispersal dynamics are key drivers of population change (Fay et al., 2021), but the mechanisms remain poorly understood. We investigate the effects of natal habitat and weather on in‐nest survival (i.e. fledging success) and local recruitment into the breeding population (i.e. juvenile survival). We also test whether pre‐ and post‐fledging conditions influence subsequent rates of local offspring recruitment into the population via a silver spoon effect.

## 2 METHODS

### 2.1 Study area

Our study was conducted between 2013 and 2022 at RSPB Geltsdale nature reserve in the North Pennines in Cumbria, UK (54.9° N–2.6° S), which is jointly owned by the Royal Society for the Protection of Birds and the Weir Trust. The survey area is an ~11 km^2^ sub‐section of the reserve comprising blanket bog, heathland and acid grassland, with an altitude of 220–440 m.

### 2.2 Study species and field methods

Whinchats are short‐lived (<8 years) Afro‐Palearctic migrants, breeding in grassland habitats throughout Europe and Western Asia and migrating annually to sub‐Saharan Africa for the northern winter. Whinchats are ground nesting and usually lay a single clutch of 4–7 eggs. The incubation period is 12–14 days with young provisioned by both parents for ~13 days before fledging. Young are capable of flight 3–5 days after fledging, with a further 9–15 days spent close to their natal nest while they are still dependent on their parents for food (Collar, 2005; Tome & Denac, 2012). Post‐independence, fledglings typically remain in their natal area for 1–2 months, when they undergo a partial moult prior to southerly migration (Collar, 2005).

We began searching for nests when Whinchats arrived at Geltsdale in May, with searches performed almost daily until nesting had ceased in July. Nests were located by observing adult behaviour (male singing, nest building, guarding and incubating). Males are typically more conspicuous than females during the breeding season, so the male of a pair was usually identified first, but once a nest was located females were also identified. We visited each nest every 3–7 days, recording clutch size, brood size and to confirm number fledged (see Table 1 for definitions). Whinchat may have replacement nests if the first fails, which typically have smaller clutches (5.4 vs. 3.4: Müller et al., 2005, 6.8 vs. 5.8: Shitikov et al., 2015) and lower fledging success (Grüebler et al., 2015). In most cases, we could not reliably determine whether a nest was a first or replacement attempt, or a second nest after the success of the first. We estimated first egg laying date through back‐calculations from either observation of incomplete clutches assuming one egg is laid daily, or for nests found post‐hatching, by back‐calculating based on chick development stage assuming 14 days of incubation and a clutch size equal to brood size plus the number of unhatched eggs. All chicks were ringed with a unique combination of three colour rings and a numbered metal ring 6–8 days post‐hatching. Fledging success was usually determined from resighting of fledglings. Fledging date was estimated from chick development stage observed during nest visits. For our analyses, we used nest visit data to determine how many chicks successfully hatched and whether a nest successfully fledged at least one chick. Nest visits and bird handling were undertaken by field workers with ringing permits granted by the British Trust for Ornithology, and to minimise nest disturbance no active nest was intrusively monitored on more than four occasions.

**TABLE 1:** Term description table.

(a) Fixed effects	Description
Altitude	Integer numeric variable denoting nest elevation in metres above sea level (mean: 317; range: 222–433).
Lay date	Integer numeric variable denoting the number of days since May 1 when laying occurred (median: 27th May; range: 9th May–3rd July).
Hatch date	Integer numeric variable denoting the number of days since May 1 when hatching occurred (median: 14th June; range: 29th May–22nd July).
Clutch size	Integer numeric variable denoting the maximum number of eggs recorded in a nest (median: 6; range: 2–7).
Brood size	Maximum number of chicks observed within the nest (median: 5; range 1–7).
Vegetation cover	Integer numeric variable denoting the percentage cover within 5, 50 and 200 m of the nests of the following vegetation, modelled as separate variables: Bracken (*Pteridium* sp.), Tree scrub (e.g. *Crataegus* sp.), Tufted hair grass (*Deschampsia caespitosa*), Purple moor grass (*Molinia caerulea*), Rush (*Juncus* sp.), Bilberry etc. (*Vaccinium myrtillus*), Heather (*Calluna vulgaris*) and Other grass (e.g. *Holcus lanatus*). Tufted hair grass and purple moor grass were recorded separately to other grass species as these can form dominant patches. Means and ranges available in supplementary material Table S1.
Nest substrate	Factor denoting the primary plant substrate upon which the nest was built. Levels: Bracken, Heather, Herbs, Purple moor grass, Moss, Bilberry and Other grass.
Number of trees	5 m radius: integer numeric variable denoting the number of trees within 5 m of the nest (median: 0; range: 0–25). 50 and 200 m radius: factor denoting the number of trees within 50 and 200 m of the nest, respectively. Binned accordingly: 0, 1–25, 26–50, 51–75, 76–100, 101–150, 151–200, 201+.
Presence of features	Binary factors denoting the presence of features within 5, 50 and 200 m of the nest. Features: Ditch/stream, Fence post, Overhead wire, Path, Boulder & Wall.
Mean temp. in‐nest	Continuous numeric variable denoting the mean temperature (°C) for each nest during the ~13‐day between hatching and fledging (mean: 12.7; range: 9.6–17.1).
Mean temp. post‐fledging (40 & 50 days)	Continuous numeric variable denoting the mean temperature (°C) during 40‐ and 50‐days following fledging (40 days mean: 13.8; range: 11.9–15.6, 50 days mean: 13.7; range: 11.9–15.3).
Total rainfall in‐nest	Continuous numeric variable denoting the total rainfall (mm), during the ~13‐days between hatching and fledging (mean: 35.9; range: 0.00–98.8).
Total rainfall post‐fledging (40 & 50 days)	Continuous numeric variable denoting the total rainfall (mm) during 40‐ and 50‐days following fledging (40 day mean: 158; range: 34.8–294, 50 day mean: 210; range: 62.5–347).
(b) Response variables	Description
Nest success	Binary factor denoting whether a nest successfully fledged at least one chick. Modelled with a binomial distribution.
Local recruitment	Binary factor designating whether at least one fledgling was resighted on their natal site in subsequent years. Modelled with a binomial distribution.
Proportion recruited	Number of resighted fledglings as a proportion of the total fledged per nest. Modelled with a binomial distribution (mean: 0.25; range 0–1).
(c) Random effect	Description
Year	Unique factor designating the year that a nest was sampled. Used throughout to account for between year variation in offspring survival.

*Note*: Details of fixed effects (a), response variables (b), and random effects (c) used throughout analysis.

Colour marked Whinchat fledglings were monitored in the year of fledging until autumn departure and were then searched for in the adult breeding population in subsequent years. Searches were made almost daily from late April to early September in all years 2013–2022. Typically, multiple observers independently surveyed the whole study area almost daily from May to July of each year. Late in the season fledglings and adults congregated to moult in certain areas, and these hotspots were surveyed more frequently in August and September.

Despite rigorous and frequent searches of the field site, some nesting attempts would inevitably have been missed. Because failed nests are active for a shorter period than successful nests, they are more likely to be missed, so a direct estimate of nest success rates from failed versus fledged nests may overestimate fledging success. To account for this, we performed a nest survival analysis (Dinsmore et al., 2002; Mayfield, 1975), which estimated the probability of nest survival from the total number of days that each nest survived (i.e. exposure days). Using this approach, we estimated a nest survival rate of 73.3%, which is slightly lower than the direct estimate from our data (80.6%). However, this is unlikely to affect our assessment of the systematic factors influencing nest survival unless these factors also influenced the likelihood of observers finding a nest. Given that nests were usually found by locating calling adults, we find it unlikely that any of the key factors we investigate (e.g. vegetation, weather) should affect the likelihood that a nest failed prior to being located. For further details on survival analysis see supplementary material.

#### 2.2.1 Vegetation and habitat sampling

The vegetation substrate on which a nest was built and the vegetation within 5 m, 50 m and 200 m radii of each nest were recorded between May–July of each year 2013–2014 and 2017–2019 after each nest had concluded. Radii boundaries were first marked, then observers measured the total area occupied by each individual vegetation type within this area, as follows: Bracken (*Pteridium* sp.), Tree scrub (e.g. *Crataegus* sp.), Tufted hair grass (*Deschampsia caespitosa*), Purple moor grass (*Molinia caerulea*), Rush (*Juncus* sp.), Bilberry etc. (*Vaccinium myrtillus*), Heather (*Calluna vulgaris*) and other grass (e.g. *Holcus lanatus*). These measures were then used to calculate the relative percentage cover by each vegetation type within each area. The number of trees and presence or absence of key features (e.g. fence post, wall) was also recorded within each radius; for the 5 m radius the number of trees was counted exactly, whereas for 50 m and 200 m radii the number of trees were estimated and binned. For further details on habitat sampling, see Table 1 and Table S1 (Appendix S1). Additionally, the elevation at which each nest was located was recorded.

#### 2.2.2 Weather data

To determine the effect of environmental conditions pre‐ and post‐fledging we estimated relevant time windows for in‐nest and post‐fledging periods for each nest. The in‐nest period, when chicks are flightless and dependent on parents for food, was defined as the period between the day of hatching and day of fledging. To account for variation of in‐nest stage duration such as from the shorter periods of failed nests, this period was standardised as the median 13‐days post‐hatching in all cases (Figure S1). The post‐fledging period covers from when initially flightless chicks leave the nest until migratory dispersal. Upon fledging, Whinchat remain within 5–10 m of their natal nest for 3–5 days when mortality risk is most acute (Tome & Denac, 2012), then typically spend a further 10–12 days within 50–75 m of their nest before increasing their range to >200 m for the remainder of the post‐fledging period (Tome & Denac, 2012). To estimate dispersal date, an analysis of the final observation dates of fledglings at Geltsdale in the year of fledging indicated large drop‐offs at 40‐ and 50‐days post‐fledging, with few observed after 50 days, suggesting most have either dispersed or died 50 days after fledging (Figure S2). To minimise estimation error, we used two different period lengths, 40‐ and 50‐days after fledging; however due to high correlation between these data, no final model included both post‐fledging period lengths. Whilst our analysis cannot distinguish post‐fledging mortality from mortality during subsequent stages of the annual cycle, we aimed to investigate links between post‐fledging conditions and survival to recruitment as these conditions may impact recruitment directly via mortality during the post‐fledging period, and indirectly by influencing subsequent survival in later stages of the annual cycle.

We downloaded data on daily interpolated maximum and minimum temperature and total precipitation for the 5 × 5 km square encompassing Geltsdale (Eastings: 36°00′00″–36°50′00″; Northings: 55°50′00″–56°00′00″) for June–September each year from CEDA (Hollis et al., 2018). We then averaged the daily maximum and minimum temperature and total rainfall values for the two respective survival stage periods. Descriptions of all fixed effects (Table 1a), response variables (Table 1b) and random effects (Table 1c) are available in Table 1.

### 2.3 Analysis

All analyses were performed using R 4.0.2 (R Core Team, 2020). Weather data were extracted and analysed using the packages raster (Hijmans, 2022), ncdf4 (Pierce, 2021) and rgdal (Bivand et al., 2022). Data were visualised using the ggplot2 (Wickham, 2016) and cowplot (Wilke, 2020) packages. All generalised linear mixed effects models (GLMMs) were built using the lme4 package (Bates et al., 2015) and analysed using lmerTest (Kuznetsova et al., 2017).

Because vegetation data was sampled during a subset of years (2013–2014 & 2017–2019), but weather and life history trait data were available for all years (2013–2021), we performed two analyses for each response variable, one using all 9 years data without vegetation variables (‘all years’), and another for the 5‐year subset with all variables including vegetation (‘vegetation years’).

Vegetation sampling produced >40 individual variables, therefore, to avoid overfitting we performed a two‐stage modelling approach first used and validated, by Pearce‐Higgins et al. (2009) (used also in: Dormann et al., 2013; Stanbury et al., 2022) to determine which explanatory variables to include in the final stage of analysis for ‘vegetation years’ models. First, a series of single‐term GLMMs were run and the effect of each term on a given response variable was assessed individually, with explanatory variables *p* ≤ .10 considered for inclusion in subsequent models (see Tables S2–S4). Second, two‐term GLMMs were used to assess which continuous variables should be included as quadratic variables. Any quadratic variables with *p* ≤ .10 were considered for inclusion in subsequent models as both quadratic and linear variables. Finally, these variables were checked for correlations, and where two variables were highly correlated with each other (Spearman rank correlation coefficient, |*r*_s_| ≥ .70, Dormann et al., 2013) the more significant predictor (i.e. the smaller *p*‐value) was included in the full model (Tables S5–S6). This process was repeated for each response variable. For the analysis of data from all years, all weather and life history variables were included in the full model as both linear and quadratic terms (Tables S7–S9) unless highly correlated (|*r*_s_| ≥ .70) with a more significant term (Tables S10–S11).

Once the number of explanatory variables was reduced, we fitted GLMMs by performing stepwise elimination by iteratively removing the term with the largest *p*‐value and then refitted the model until the Minimum Adequate Model (MAM) remained (all variables *p* < .05). In total, we ran GLMMs for four response variables with ‘Year’ as a random effect for both the full sample of all years and for the vegetation years, all using the reduced set of predictors as fixed effects and including year as a random effect. Full details of all model structures, their response variables, fixed effects (full model and MAM), error distributions and sample sizes are available in Table 2, full model outputs are available in Tables S12–S17.

**TABLE 2:** Model structures for generalised linear mixed effects models.

Response variable	Data used	Explanatory variables	Sample size (nests)
Nest success	Vegetation years	**Brood size** + **Bracken 50 m** + **(Bracken 50 m)** ^**2**^ + Tree scrub 5 m + Tree scrub 200 m + Heather 50 m + (Heather 50 m)^2^ + Ditch/stream 5 m + Overhead wires 200 m + Path 50 m + **Mean temp. in‐nest** + **Mean temp. in‐nest** ^**2**^	143
	All years	**Altitude** + Altitude^2^ + Lay date + Lay date^2^ + **Brood size** + Brood size^2^ + Mean temp. in‐nest + Mean temp. in‐nest^2^ + Total rainfall in‐nest + Total rainfall in‐nest^2^	247
Local recruitment	Vegetation years, fledged only	Altitude + Altitude^2^ + Lay date + **Brood size** + Bracken 200 m + Rush 50 m + Rush 200 m + Bilberry 5 m + Heather 50 m + **Heather 200 m** + Path 5 m + Mean temp. in‐nest + Mean temp 50 days post‐fledge	118
	All years, fledged only	Altitude + Altitude^2^ + Hatch date + Hatch date^2^ + **Brood size** + Brood size^2^ + Mean temp. in‐nest + Mean temp. in‐nest^2^ + Total rainfall in‐nest + Total rainfall in‐nest^2^ + Mean temp. 40 days post‐fledge + Mean temp. 40 days post‐fledge^2^ + Total rainfall 40 days post‐fledge + Total rainfall 40 days post‐fledge^2^	197
Proportion recruited	Vegetation years, fledged only	**Brood size** + **Tree scrub 200 m** + **(Tree scrub 200 m)** ^**2**^ + Other grass 200 m + Rush 200 m + **Heather 50 m** + **(Heather 50 m)** ^**2**^ + Fence posts 5 m + Path 200 m + **Total rainfall in‐nest** + Total rainfall in‐nest^2^ + Mean temp. 50 days post‐fledge	118
	All years, fledged only	Altitude + Altitude^2^ + Lay date + Lay date^2^ + Clutch size + Clutch size^2^ + Mean temp. in‐nest + Mean temp. in‐nest^2^ + Total rainfall in‐nest + Total rainfall in‐nest^2^ + **Mean temp. 40 days post‐fledge** + **Mean temp. 40 days post‐fledge** ^**2**^ + Total rainfall 40 days post‐fledge + Total rainfall 40 days post‐fledge^2^	197

*Note*: Life history traits and weather variables were collected for all years (2013–2021), and vegetation characteristics were collected for a subset of years (2013–2014 & 2017–2019). Model terms were selected using a two‐stage modelling approach, as follows: each term was modelled in a single term model and terms with *p*‐values < .10 were considered for inclusion in the full model unless highly correlated (|*r*_s_| > .70) with another term. Stepwise elimination of terms from this full model was then performed until only significant terms remained, leaving a minimum adequate model (MAM, terms in bold). All models were run including the random effect ‘Year’. Local recruitment refers to whether a nest successfully had ≥1 fledgling resighted in subsequent years. Sample sizes for resighting analysis are smaller than analysis of fledging success because two nests were omitted due to missing fledge date, with weather during the post‐fledging period not possible to use. All models were fitted with a binomial distribution.

## 3 RESULTS

### 3.1 Nest success

**FIGURE 1:**
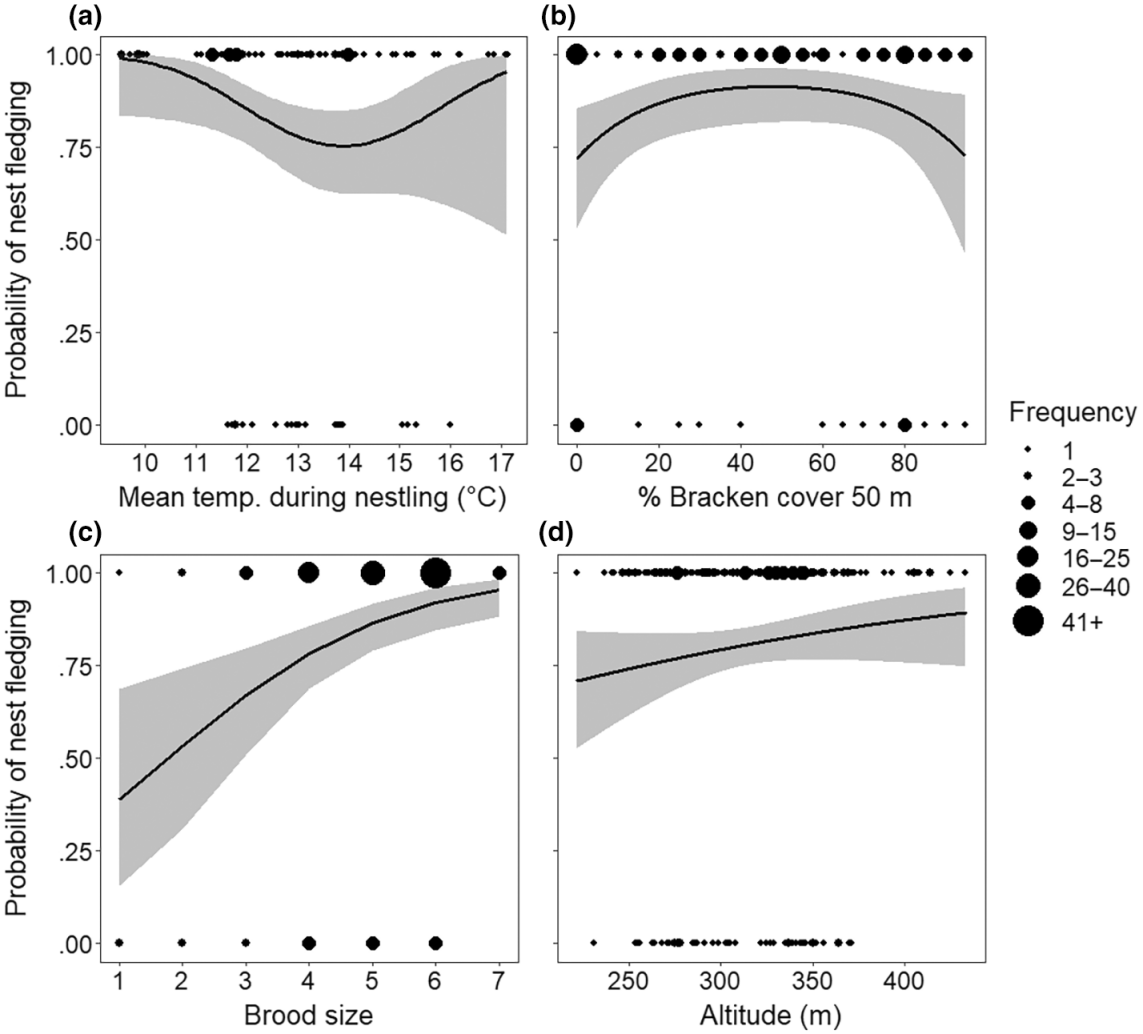
The effect of key variables on fledging success. The probability of a nest fledging at least one chick against: (a) mean temperature during the in‐nest stage (°C), from years when vegetation data were collected (*N* = 143 nests); (b) percentage bracken cover within 50 m of the nest, from years when vegetation data were collected (*N* = 143); (c) brood size, from all years (*N* = 247); and (d) altitude, from all years (*N* = 247). Predicted relationships (± 95% CI) are fitted from generalised linear mixed effects models, see Table 3.

Overall, survival analysis estimated a nest success rate of 73.3%, though within our sample 199/247 (80.6%) of broods successfully fledged at least one chick and successful nests, on average, fledged 4.88 ± 0.10 (SE) chicks. For the vegetation years, nest success increased with brood size (GLMM: *p* < .001, Table 3a), was highest at high (17°C) and low (10°C) temperature extremes experienced during the in‐nest period (GLMM quadratic relationship: *p* = .019, Table 3a, Figure 1a), and was highest where Bracken *Pteridium aquilinum* cover within 50 m of the nest was 40%–60% (GLMM quadratic relationship: *p* = .032, Table 3a, Figure 1b). There was a marginally non‐significant association with cover of tree scrub within 200 m of the nest (GLMM: *p* = .056, Table S12) and during stepwise elimination, removal of either the Bracken or Tree scrub term resulted in the other being significant, though neither were significant when modelled together. The all‐years MAM (*N* = 247) also found that fledging success increased with brood size (GLMM: *p* < .001, Table 3b, Figure 1c), and found a significant positive effect of altitude on nest success (GLMM: *p* = .024, Table 3b, Figure 1d).

**TABLE 3:** Generalised linear mixed effects models outputs for variables influencing whether a brood successfully fledged at least one chick.

Fixed effects	Estimates ± SE	df	*χ* ^2^	*p*‐Value
(a) Nest success vegetation years				
Brood size	0.92 ± 0.26	1, 143	12.37	<.001
Bracken 50 m	2.28 ± 0.93	1, 143	6.06	.014
(Bracken 50 m)^2^	−2.05 ± 0.95	1, 143	4.61	.032
Mean temp. nesting	−11.37 ± 4.84	1, 143	5.51	.019
Mean temp. nesting^2^	11.03 ± 4.70	1, 143	5.52	.019
(b) Nest success all years				
Altitude	0.42 ± 0.19	1, 247	5.06	.024
Brood size	0.56 ± 0.17	1, 247	10.63	.001

*Note*: Analysis performed on data from years with vegetation and landmark features sampled (143 out of 247 broods) and from all years (*N* = 247). Outputs shown are from the minimum adequate model (*p* ≥ .05). Full model outputs available from Tables S12–S13.

### 3.2 Local recruitment

A total of 1193 young were marked as nestlings prior to fledging with 436 of these observed a total of 993 times in the same year of fledging, and 242 observed at least once after the year of fledging.

#### 3.2.1 Recruitment probability

**FIGURE 2:**
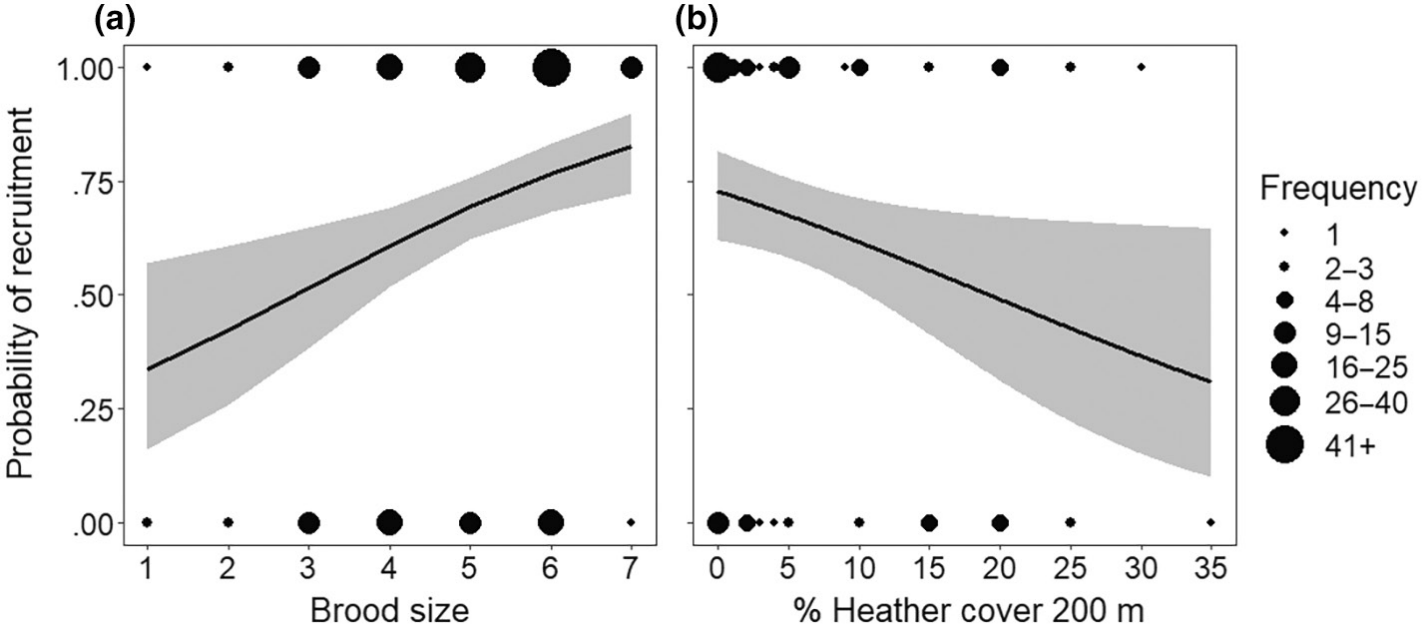
The effect of key variables on the likelihood of a nest producing one recruit. The probability of at least one fledgling from a successful nest being resighted in subsequent years against (a) brood size, from all years (*N* = 197 nests); and (b) percentage Heather cover within 200 m of the nest, from years when vegetation data were collected (*N* = 118 nests). Predicted relationships (± 95% CI) are fitted from generalised linear mixed effects models, see Table 4.

Across all years, 68.5% (135/197) of successful nests had at least one fledgling observed in subsequent years, our proxy for local recruitment. The vegetation years MAM revealed that the probability of a successful nest producing at least one local recruit increased with brood size (GLMM: *p* < .001, Table 4a, Figure 2a) and declined with increasing Heather *Calluna vulgaris* cover within 200 m of the nest (GLMM: *p* = .013, Table 4a, Figure 2b). The all‐years MAM also revealed that, as expected, the probability of a successful nest producing at least one local recruit also increased with brood size (GLMM: *p* < .001, Table 4b, Figure 2a), and indicated a near significant trend of recruitment declining with later hatch date (GLMM: *p* = .054, Table S15).

**TABLE 4:** Generalised linear mixed effects models outputs for variables influencing whether a nest had at least one fledgling recruited.

Fixed effects	Estimates ± SE	df	*χ* ^2^	*p*‐Value
(a) Local recruitment vegetation years				
Brood size	0.73 ± 0.22	1, 118	11.20	<.001
Heather 200 m	−0.50 ± 0.20	1, 118	6.12	.013
(b) Local recruitment all years				
Brood size	0.50 ± 0.16	1, 197	9.45	.002

*Note*: Analysis performed on data from years with vegetation and landmark features sampled (143 out of 247 broods) and all years (*N* = 247). Outputs shown are from the minimum adequate model (*p* ≥ .05). Full model outputs available from Tables S14–S15.

#### 3.2.2 Proportion recruited

**FIGURE 3:**
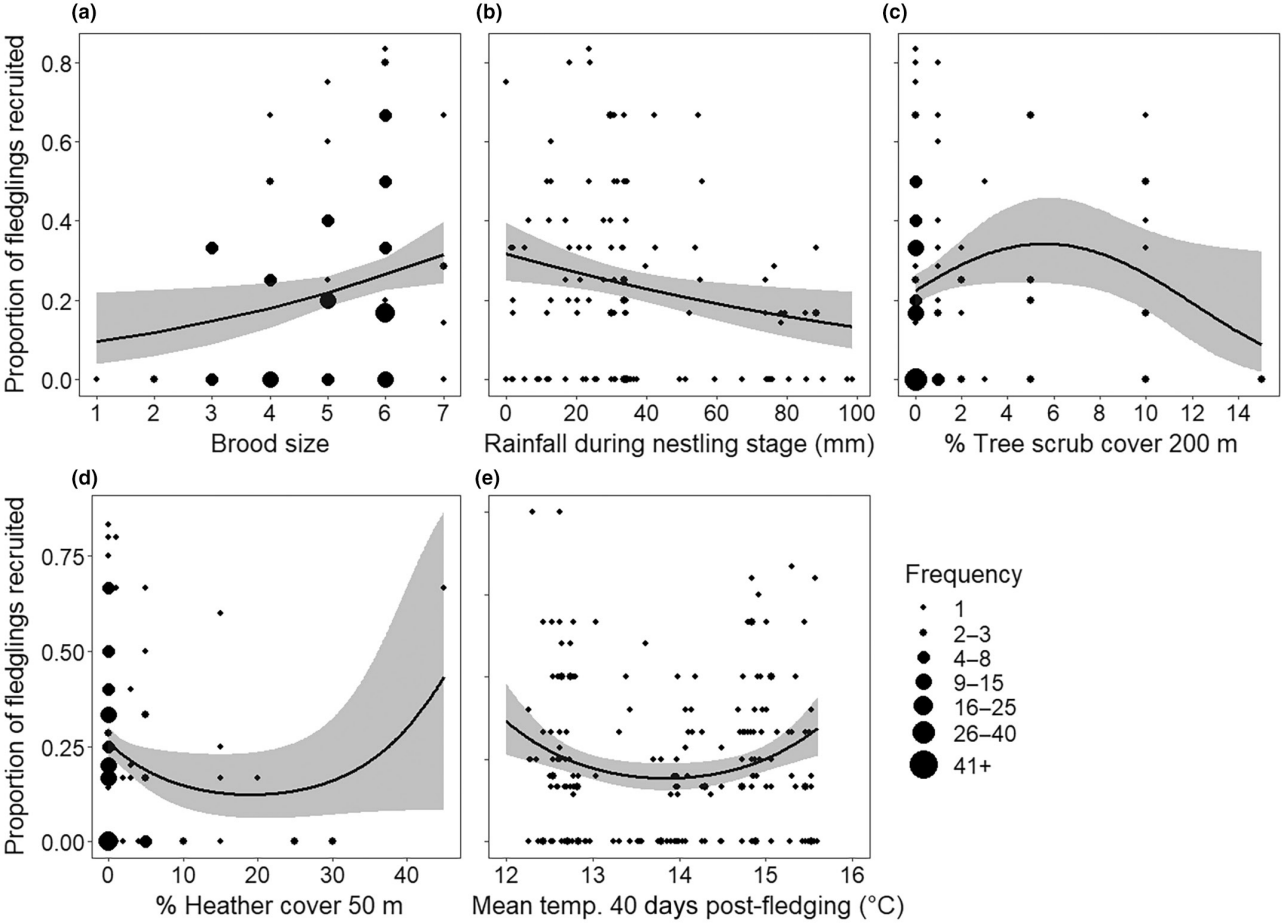
The effect of key variables on the proportion of fledglings recruited. The proportion of fledglings from successful nests which were resighted in subsequent years against (a) brood size, from years when vegetation data were collected (*N* = 118 nests); (b) total rainfall during the in‐nest stage, from years when vegetation data were collected (*N* = 118); (c) percentage Tree scrub cover within 200 m of the nest, from years when vegetation data were collected (*N* = 118 nests); (d) percentage Heather cover within 50 m of the nest, from years when vegetation data were collected (*N* = 118 nests); and (e) mean temperature for 40 days following fledging, all years (*N* = 197 nests). Predicted relationships (± 95% CI) are fitted from generalised linear mixed effects models, see Table 5.

Across all years, 1.76 ± 0.09 fledglings per successful nest locally recruited, with 24.3% (237/976) of fledglings recruited in total. The vegetation years MAM (*N* = 118 nests) indicated that the proportion of fledglings from successful nests recruited locally increased with brood size (GLMM: *p* < .001, Table 5a, Figure 3a), decreased with rainfall during the in‐nest stage (GLMM: *p* = .001, Table 5a, Figure 3b), was highest at mid‐range values of Tree scrub cover (2%–10%) within 200 m of the nest (GLMM quadratic relationship: *p* = .021, Table 5a, Figure 3c) and was higher at either high (>30% cover) or low (<10% cover) values of Heather cover within 50 m of the nest (GLMM quadratic relationship: *p* = .004, Table 5a, Figure 3d). However, the quadratic nature of this latter trend appeared to be driven by a single outlier, the removal of this point suggested that proportion recruited was linearly negatively related to Heather cover. The all‐years MAM (*N* = 197) revealed that local recruitment was highest at both temperature extremes (<12°C and >16°C) experienced during the 40‐day post‐fledging period (GLMM quadratic relationship: *p* = .005, Table 5b, Figure 3e).

**TABLE 5:** Generalised linear mixed effects models outputs for variables influencing the proportion of fledglings recruited per nest.

Fixed effects	Estimates ± SE	df	*χ* ^2^	*p*‐Value
(a) Proportion recruited vegetation years				
Brood size	0.41 ± 0.15	1, 118	7.47	.006
Tree scrub 200 m	0.88 ± 0.36	1, 118	5.97	.015
(Tree scrub 200 m)^2^	−0.90 ± 0.39	1, 118	5.32	.021
Heather 50 m	−0.94 ± 0.32	1, 118	8.53	.003
(Heather 50 m)^2^	0.91 ± 0.31	1, 118	8.47	.004
Total rainfall nesting	−0.41 ± 0.13	1, 118	10.11	.001
(b) Proportion recruited all years				
Mean temp. 40 days post‐fledge	−7.70 ± 2.71	1, 197	8.07	.004
Mean temp 40 days post‐fledge^2^	7.69 ± 2.71	1, 197	8.05	.005

*Note*: Analysis performed on data from years with vegetation and landmark features sampled, and nests which successfully fledged (143 out of 247 broods) and nests which fledged from all years (*N* = 247). Outputs shown are from the minimum adequate model (*p* ≥ .05). Full model outputs available from Tables S16–S17.

## 4 DISCUSSION

In summary, we found that larger Whinchat broods: (i) occurred most in the early‐mid stage of the breeding season, (ii) were more likely to fledge and (iii) showed higher rates of apparent survival to recruitment. Importantly, fledglings that experienced higher rainfall prior to fledging had lower apparent survival to recruitment, suggesting a silver spoon effect where conditions experienced in‐nest influenced subsequent local recruitment probability. Nests that experienced temperature extremes prior to fledging were more likely to fledge, and fledglings that experienced temperature extremes in the post‐fledging period had higher apparent survival to recruitment. Additionally, nests with mid‐range Bracken cover (50%) were most likely to fledge, with other microhabitat features influencing apparent survival to recruitment to a lesser extent.

### 4.1 Life history traits

In addition to producing more fledglings, larger broods were also more likely to successfully fledge. For migratory birds, favourable conditions in non‐breeding locations are often associated with earlier arrival to breeding sites, earlier egg laying and higher productivity (e.g. Ockendon et al., 2013), including for Whinchat (Grüebler et al., 2015; Müller et al., 2005; Shitikov et al., 2015). A relationship between the timing of breeding and clutch size, and thus brood size, is well established in Whinchat (Fuller & Glue, 1977; Grudinskaya et al., 2022), including in our study, where brood size peaked for nests that hatched in the early–middle period of the breeding season, then declined over time (Table S18, Figure S3). This effect may be partly due to parental fitness or experience because higher quality and/or more experienced individuals may arrive earlier to the breeding site, produce larger broods, and exhibit higher reproductive success. Additionally, Whinchat may produce replacement broods in the event of nest failure, which relative to their first, typically have smaller clutches (5.4 vs. 3.4: Müller et al., 2005, 6.8 vs. 5.8: Shitikov et al., 2015) and lower fledging success (Grüebler et al., 2015). Also, although not well documented for Whinchat, it is occasionally possible for early breeding pairs to have a second brood after the success of their first. We confirmed two instances of this in 2016 where both parents were colour marked, and suspected a further six instances from the same year where males were associated with a second nest after the success of their first. However, in most cases we were unable to determine with certainty whether a nest was a replacement due to the low proportion of colour marked parents and high divorce rate of Whinchats after nest failure (52%; Grüebler et al., 2015), though we suspected low rates of renesting in our population. None the less, renesting likely contributed to the peak in brood size in the early‐mid stage of the season (two apparent peaks in nesting activity; Figure S3), and to the positive relationship between brood size and nest success. In a previous study, lower survival of replacement Whinchat broods was a consequence of timing differences within the season rather than intrinsic differences between first and replacement nests (Grüebler et al., 2015). Therefore, Whinchat broods should experience the same survival challenges regardless of parental quality, experience or whether they were replacement nests, so these results still require a mechanism by which larger or later broods have lower reproductive success. Daily in‐nest survival rates are often higher in earlier broods (Low & Pärt, 2009), possibly due to seasonal increases in predation threat (Hatchwell, 1991; Verhulst & Nilsson, 2007). Earlier fledging can lower rates of post‐fledging predation (Naef‐Daenzer & Grüebler, 2016; Verhulst & Nilsson, 2007), and pre‐fledging conditions can influence post‐fledging survival; with fledglings from earlier broods seemingly less susceptible to mortality in subsequent stages (Naef‐Daenzer & Grüebler, 2016). Furthermore, higher success for larger broods is unlikely to be due to buffering against complete failure due to cumulative events of mortality, as Whinchat experience very low rates of partial brood failure (7.3% in our study; 3% in Border, Henderson, Ash, et al., 2017), meaning that the number of fledglings was highly correlated with brood size (*r*_s_ = .827).

We used resighting rates of fledglings returning as adults to estimate apparent survival to recruitment, so we could not distinguish mortality from dispersal to outside our study area. Therefore, we cannot exclude the possibility that natal conditions, for example, brood size, fledging date, or whether they were from a replacement brood, differentially affect dispersal movements. However, we recorded a high rate of nestlings recruiting into our study population (20%), which coupled with low natal dispersal distance (Shitikov et al., 2012) and high adult high site fidelity in Whinchat (Blackburn & Cresswell, 2016), gives confidence to our findings on recruitment rate. For example, we found that fledglings from larger broods had a higher apparent recruitment rate. Clutch size, and hence brood size, is predominantly related to pre‐laying environmental conditions experienced in the breeding area (Haywood & Perrins, 1992), breeding phenology (Müller et al., 2005; Shitikov et al., 2015) and parental fitness (Smith & Moore, 2003). In Whinchat, a carry‐over effect could influence breeding phenology, for example, a higher mass upon departure from African non‐breeding sites can correspond with earlier departure (Risely et al., 2015). We suggest that the observed positive effect of brood size on reproductive success is driven largely by higher reproductive success for earlier timed nests, which could be mediated by seasonal variation in predation risk and/or variation in the length of the post‐fledging period prior to migration. However, we note that parental quality or experience, differences between first and replacement nests, and carry‐over effects from non‐breeding site conditions can also influence reproductive success, both directly and indirectly via the timing of breeding. It is also possible that positive selection for earlier breeding may in part also arise from higher rates of recruitment by young from earlier nests as an endogenous mechanism of heritable timing (Akresh et al., 2021; Bazzi et al., 2016; Saino et al., 2015; Sosnovcová et al., 2018).

### 4.2 Weather

Temperature extremes (i.e. <13°C or >15°C) experienced during the in‐nest period resulted in a higher likelihood of fledging, and a higher apparent recruitment rate. This is surprising given that daily nest survival decreases linearly with temperature for many passerines (e.g. Low & Pärt, 2009; Tome et al., 2020). Rates of nest predation can also increase (Cox, Thompson, & Reidy, 2013; Cox, Thompson, Reidy, et al., 2013) or decrease (Skagen & Yackel Adams, 2012) with temperature. Main predators of young Whinchats are small mammals (e.g. red fox, *Vulpes vulpes*), corvids (e.g. magpies, *Pica pica*) and raptors (e.g. common buzzard, *Buteo buteo*) (Frankiewicz, 2008; Tome & Denac, 2012). Exactly why mid‐range temperatures predict high in‐nest and post‐fledging mortality is not clear, but we speculate that this may be mediated by variation in predator activity or efficiency of predator foraging at different temperature extremes. For example, at cold temperatures chicks may require more provisioning to maintain homeostasis and thus may beg louder or more frequently (Kilner & Johnstone, 1997) potentially attracting auditory predators. Likewise, during the post‐fledging period recently independent fledglings may have to take more risks when foraging at low temperatures (Hilton et al., 1999). Whereas, at high temperatures, favourable temperature and/or light conditions may increase hunting activity of predators (Cox, Thompson, & Reidy, 2013).

Fledglings that experienced higher rainfall prior to fledging had lower apparent survival to recruitment. Weather conditions, especially rainfall, are known to increase pre‐ and post‐fledging mortality of passerines, but in our study rainfall prior to fledging influenced apparent recruitment, suggesting that conditions Whinchat fledglings experienced in‐nest influenced survival during subsequent stages. However, as we could not determine whether this effect was mediated by a change in mortality during the post‐fledging period (i.e. intra‐seasonal) or during subsequent stages of the annual cycle (i.e. inter‐seasonal) we are unable to conclude whether this was a true carry‐over effect or just a silver spoon effect. Similar effects have been found in other passerines, with better condition fledglings experiencing higher post‐fledging survival (Naef‐Daenzer & Grüebler, 2016; Vitz & Rodewald, 2011). High rainfall prior to fledging may increase thermoregulatory costs to nestlings (Visser et al., 1998) or reduce prey availability for parents provisioning their offspring and hence slow provisioning rate, thus diminishing offspring condition (Öberg et al., 2015; Radford et al., 2010).

### 4.3 Vegetation

The only vegetation term to significantly impact nest success was Bracken cover within 50 m of the nest, which had a quadratic relationship with likelihood of fledging, with success highest at around 50% cover. It is perhaps unsurprising that Bracken was associated with nest success, given that Whinchat breeding in the UK frequently associate with high proportions of Bracken cover (Allen, 1995; Pearce‐Higgins & Grant, 2006; Stanbury et al., 2022). However, the quadratic nature of the relationship is interesting, with fledging success declining above 50% Bracken cover. Whinchats preferentially nest in areas with high structural vegetation diversity (Border, Henderson, Redhead, et al., 2017; Douglas et al., 2017; Fischer et al., 2013), which are associated with greater quality, diversity and abundance of invertebrate prey (Britschgi et al., 2006; Evans et al., 2015) but avoid foraging in areas with high Bracken cover (Murray et al., 2016). Therefore, a trade‐off may exist between nest concealment offered by high Bracken cover and the low foraging efficiency of Bracken monocultures.

Tree scrub cover within 200 m of the nest also showed a marginally non‐significant negative trend with likelihood of fledging, and the removal of the Bracken 50 m term from the model resulted in Tree scrub cover being significant. We attribute this to the negative correlation between Bracken and Tree scrub cover (*r*_s_ = −.41, Table S5). Similarly, analysis of all years, which did not include vegetation measures, found that the likelihood of fledging increased with altitude, which we suggest is driven by a weak positive correlation between altitude and Bracken cover (50 m; *r*_s_ = .38, Table S5).

A review by Cox et al. (2014) found that local habitat influenced post‐fledging survival in most studies of passerine birds. Within 200 m of the nest, we found that rates of apparent recruitment decreased with Heather cover (with one outlier) and had a quadratic relationship with Tree scrub cover, peaking around 6%. Vegetation cover around the nest may affect apparent survival to recruitment by influencing predation risk during the vulnerable post‐fledging period and/or through offspring condition related to territory scale invertebrate prey availability during the in‐nest or post‐fledging period (Naef‐Daenzer & Grüebler, 2016, Vitz & Rodewald, 2011), which could influence fledgling condition and thus influence survival at subsequent stages.

## 5 CONCLUSION

Our study provides evidence of potential silver spoon effects from natal brood size and rainfall during the in‐nest period to subsequent survival to recruitment into the local breeding population in a migratory passerine. However, it is not known whether this effect was mediated by a change in survival during the post‐fledging period (i.e. intra‐seasonal) or during subsequent stages (i.e. inter‐season), thus we cannot determine whether this is an example of a true carry‐over effect. Similar effects detected in other migratory birds mostly occur within an annual cycle, linking non‐breeding or migration conditions to reproduction within the same annual cycle. However, we appear to detect some sustained influence of early‐life effects on mortality rate during subsequent stages, which could be especially crucial for maintaining population persistence (i.e. a silver spoon effect). This highlights the importance of identifying the drivers of juvenile survival and local recruitment for declining migratory birds, and the role of early‐life conditions, to better understand the mechanisms.

## AUTHOR CONTRIBUTIONS

**Chay Halliwell:** Conceptualization (lead); data curation (lead); formal analysis (lead); methodology (equal); project administration (supporting); validation (equal); visualization (equal); writing – original draft (lead); writing – review and editing (equal). **Martin Ketcher:** Data curation (equal); investigation (equal); methodology (equal); writing – review and editing (supporting). **Amanda Proud:** Data curation (supporting); investigation (equal); methodology (equal); writing – review and editing (supporting). **Stephen Westerberg:** Data curation (equal); investigation (equal); methodology (equal); project administration (lead); writing – review and editing (supporting). **David J. T. Douglas:** Conceptualization (equal); methodology (equal); project administration (equal); supervision (equal); validation (equal); visualization (equal); writing – review and editing (equal). **Malcolm D. Burgess:** Conceptualization (equal); methodology (equal); project administration (equal); supervision (equal); validation (equal); visualization (equal); writing – review and editing (equal).

## FUNDING INFORMATION

This work was supported by the Natural Environment Research Council (NE/S00713X/1).

## CONFLICT OF INTEREST STATEMENT

The authors declare no conflict of interest.

### OPEN RESEARCH BADGES

This article has earned Open Data and Open Materials badges. Data and materials are available at https://doi.org/doi:10.5061/dryad.msbcc2g3p.

## Supporting information


Appendix S1

## Data Availability

All data and code used in this analysis are available from https://doi.org/doi:10.5061/dryad.msbcc2g3p.

## References

[ece310346-bib-0001] Akresh, M. E. , King, D. I. , & Marra, P. P. (2021). Hatching date influences winter habitat occupancy: Examining seasonal interactions across the full annual cycle in a migratory songbird. Ecology and Evolution, 11, 9241–9253. 10.1002/ece3.7500 34306620 PMC8293775

[ece310346-bib-0002] Allen, D. S. (1995). Habitat selection by whinchats: A case for bracken in the uplands? In D. B. A. Thompson , A. J. Hester , & M. B. Usher (Eds.), Heaths and moorland: Cultural landscapes (pp. 200–205). HMSO.

[ece310346-bib-0005] Bates, D. , Maechler, M. , Bolker, B. , & Walker, S. (2015). Fitting linear mixed‐effects models using lme4. Journal of Statistical Software, 67, 1–48. 10.48550/arXiv.1406.5823

[ece310346-bib-0006] Bazzi, G. , Cecere, J. G. , Caprioli, M. , Gatti, E. , Gianfranceschi, L. , Podofillini, S. , Possenti, C. D. , Ambrosini, R. , Saino, N. , Spina, F. , & Rubolini, D. (2016). Clock gene polymorphism, migratory behaviour and geographic distribution: A comparative study of trans‐Saharan migratory birds. Molecular Ecology, 25, 6077–6091. 10.1111/mec.13913 27862517

[ece310346-bib-0009] Bivand, R. , Keitt, T. , & Rowlingson, B. (2022). Rgdal: Bindings for the 'Geospatial' data abstraction library . R Package Version 1.5‐32. https://CRAN.R‐project.org/package=rgdal

[ece310346-bib-0011] Blackburn, E. , & Cresswell, W. (2016). High winter site fidelity in a long‐distance migrant: Implications for wintering ecology and survival estimates. Journal of Ornithology, 157, 93–108. 10.1007/s10336-015-1252-z

[ece310346-bib-0012] Border, J. A. , Henderson, I. G. , Ash, D. , & Hartley, I. R. (2017). Characterising demographic contributions to observed population change in a declining migrant bird. Journal of Avian Biology, 48, 1139–1149. 10.1111/jav.01305

[ece310346-bib-0013] Border, J. A. , Henderson, I. G. , Redhead, J. W. , & Hartley, I. R. (2017). Habitat selection by breeding whinchats *Saxicola rubetra* at territory and landscape scales. Ibis, 159, 139–151. 10.1111/ibi.12433

[ece310346-bib-0200] Britschgi, A. , Spaar, R. , & Arlettaz, R. (2006). Impact of grassland farming intensification on the breeding ecology of an indicator insectivorous passerine, the Whinchat *Saxicola rubetra*: Lessons for overall alpine meadowland management. Biological Conservation, 130, 193–205. 10.1007/s10336-011-0799-6

[ece310346-bib-0016] Cockburn, A. (1991). An introduction to evolutionary ecology. Blackwell Scientific Publications.

[ece310346-bib-0018] Collar, N. (2005). Whinchat. In J. del Hoyo , A. Elliott , & D. Christie (Eds.), Handbook of birds of the world (Vol. 10, p. 777). Lynx Edicions.

[ece310346-bib-0019] Cox, A. W. , Thompson, F. R., III , & Reidy, J. L. (2013). The effects of temperature on Nest predation by mammals, birds, and snakes. The Auk, 130, 784–790. 10.1525/auk.2013.13033

[ece310346-bib-0020] Cox, W. A. , Thompson, F. R., III , Cox, A. S. , & Faaborg, J. (2014). Post‐fledging survival in passerine birds and the value of post‐fledging studies to conservation. The Journal of Wildlife Management, 78, 183–193. 10.1002/jwmg.670

[ece310346-bib-0021] Cox, W. A. , Thompson, F. R., III , Reidy, J. L. , & Faaborg, J. (2013). Temperature can interact with landscape factors to affect songbird productivity. Global Change Biology, 19, 1064–1074. 10.1111/gcb.12117 23504884

[ece310346-bib-0024] Dinsmore, S. J. , White, G. C. , & Knopf, F. L. (2002). Advanced techniques for modeling avian nest survival. Ecology, 83, 3476–3488. 10.1890/0012-9658(2002)083[3476:ATFMAN]2.0.CO;2

[ece310346-bib-0026] Dormann, C. F. , Elith, J. , Bacher, S. , Buchmann, C. , Carl, G. , Carré, G. , Marquéz, J. R. G. , Gruber, B. , Lafourcade, B. , Leitão, P. J. , & Münkemüller, T. (2013). Collinearity: A review of methods to deal with it and a simulation study evaluating their performance. Ecography, 36, 27–46. 10.1111/j.1600-0587.2012.07348.x

[ece310346-bib-0027] Douglas, D. J. T. , Beresford, A. , Selvidge, J. , Garnett, S. , Buchanan, G. M. , Gullett, P. , & Grant, M. C. (2017). Changes in upland bird abundances show associations with moorland management. Bird Study, 64, 242–254. 10.1080/00063657.2017.1317326

[ece310346-bib-0029] Evans, D. M. , Villar, N. , Littlewood, N. A. , Pakeman, R. J. , Evans, S. A. , Dennis, P. , Skartveit, J. , & Redpath, S. M. (2015). The cascading impacts of livestock grazing in upland ecosystems: A 10‐year experiment. Ecosphere, 6, 1–15.

[ece310346-bib-0030] Evans, D. R. , Hobson, K. A. , Kusack, J. W. , Cadman, M. D. , Falconer, C. M. , & Mitchell, G. W. (2020). Individual condition, but not fledging phenology, carries over to affect post‐fledging survival in a Neotropical migratory songbird. Ibis, 162, 331–344. 10.1111/ibi.12727

[ece310346-bib-0031] Faaborg, J. , Holmes, R. T. , Anders, A. D. , Bildstein, K. L. , Dugger, K. M. , Gauthreaux, S. A., Jr. , Heglund, P. , Hobson, K. A. , Jahn, A. E. , Johnson, D. H. , Latta, S. C. , Levey, D. J. , Marra, P. P. , Merkord, C. L. , Nol, E. , Rothstein, S. I. , Sherry, T. W. , Sillett, T. S. , Thompson, F. R., III , & Warnock, N. (2010). Conserving migratory land birds in the New World: Do we know enough? Ecological Applications, 20, 398–418. 10.1890/09-0397.1 20405795

[ece310346-bib-0032] Fay, R. , Schaub, M. , Banik, M. V. , Border, J. A. , Henderson, I. G. , Fahl, G. , Feulner, J. , Horch, P. , Korner, F. , Müller, M. , Michel, V. , Rebstock, H. , Shitikov, D. A. , Tome, D. , Vögeli, M. , & Grüebler, M. U. (2021). Whinchat survival estimates across Europe: Can excessive adult mortality explain population declines? Animal Conservation, 24, 15–25. 10.1111/acv.12594

[ece310346-bib-0033] Finch, T. , Pearce‐Higgins, J. W. , Leech, D. I. , & Evans, K. L. (2014). Carry‐over effects from passage regions are more important than breeding climate in determining the breeding phenology and performance of three avian migrants of conservation concern. Biodiversity and Conservation, 23, 2427–2444. 10.1007/s10531-014-0731-5

[ece310346-bib-0034] Fischer, K. , Busch, R. , Fahl, G. , Kunz, M. , & Knopf, M. (2013). Habitat preferences and breeding success of whinchats (*Saxicola rubetra*) in the Westerwald mountain range. Journal of Ornithology, 154, 339–349. 10.1007/s10336-012-0898-z

[ece310346-bib-0035] Frankiewicz, J. (2008). Breeding biology and ecology of whinchat *Saxicola rubetra* on abandoned farmland of Opole Province (SW Poland). Acta Zoologica Cracoviensia‐Series A: Vertebrata, 51, 35–47. 10.3409/azc.51a_1-2.35-47

[ece310346-bib-0037] Fuller, R. J. , & Glue, D. E. (1977). The breeding biology of the stonechat and whinchat. Bird Study, 24, 215–228. 10.1080/00063657709476561

[ece310346-bib-0038] Grafen, A. (1988). On the uses of data on lifetime reproductive success. In T. H. Clutton‐Brock (Ed.), Reproductive success (pp. 454–463). University of Chicago Press.

[ece310346-bib-0039] Grudinskaya, V. , Samsonov, S. , Galkina, E. , Grabovsky, A. , Makarova, T. , Vaytina, T. , Fedotova, S. , & Shitikov, D. (2022). Effects of spring weather on laying dates, clutch size, and nest survival of ground‐nesting passerines in abandoned fields. Avian Conservation and Ecology, 17, 1–10.

[ece310346-bib-0040] Grüebler, M. U. , Schuler, H. , Spaar, R. , & Naef‐Daenzer, B. (2015). Behavioural response to anthropogenic habitat disturbance: Indirect impact of harvesting on whinchat populations in Switzerland. Biological Conservation, 186, 52–59. 10.1016/j.biocon.2015.02.031

[ece310346-bib-0041] Harrison, X. A. , Blount, J. D. , Inger, R. , Norris, D. R. , & Bearhop, S. (2011). Carry‐over effects as drivers of fitness differences in animals. Journal of Animal Ecology, 80, 4–18. 10.1111/j.1365-2656.2010.01740.x 20726924

[ece310346-bib-0042] Hatchwell, B. J. (1991). An experimental study of the effects of timing of breeding on the reproductive success of common guillemots (*Uria aalge*). Journal of Animal Ecology, 60, 721–736. 10.2307/5410

[ece310346-bib-0043] Haywood, S. , & Perrins, C. M. (1992). Is clutch size in birds affected by environmental conditions during growth? Proceedings of the Royal Society of London B: Biological Sciences, 249, 195–197. 10.1098/rspb.1992.0103 1360680

[ece310346-bib-0044] Hijmans, R. (2022). Raster: Geographic data analysis and modeling . R Package Version 3.5‐29. https://CRAN.R‐project.org/package=raster

[ece310346-bib-0045] Hilton, G. M. , Ruxton, G. D. , & Cresswell, W. (1999). Choice of foraging area with respect to predation risk in redshanks: The effects of weather and predator activity. Oikos, 87, 295–302. 10.2307/3546744

[ece310346-bib-0046] Hollis, D. , McCarthy, M. , Kendon, M. , Legg, T. , & Simpson, I. (2018). HadUK‐Grid gridded and regional average climate observations for the UK. Centre for Environmental Data Analysis. http://catalogue.ceda.ac.uk/uuid/4dc8450d889a491ebb20e724debe2dfb

[ece310346-bib-0047] Howard, C. , Stephens, P. A. , Pearce‐Higgins, J. W. , Gregory, R. D. , Butchart, S. H. M. , & Willis, S. G. (2020). Disentangling the relative roles of climate and land cover change in driving the long‐term population trends of European migratory birds. Diversity and Distributions, 26, 1442–1455. 10.1111/ddi.13144

[ece310346-bib-0048] Kilner, R. , & Johnstone, R. A. (1997). Begging the question: Are offspring solicitation behaviours signals of need? Trends in Ecology & Evolution, 12, 11–15. 10.1016/S0169-5347(96)10061-6 21237955

[ece310346-bib-0049] Kuznetsova, A. , Brockhoff, P. B. , & Christensen, R. H. B. (2017). lmerTest package: Tests in linear mixed effects models. Journal of Statistical Software, 82, 1–26. 10.18637/jss.v082.i13

[ece310346-bib-0050] Latta, S. C. , Cabezas, S. , Mejia, D. A. , Paulino, M. M. , Almonte, H. , Miller‐Butterworth, C. M. , & Bortolotti, G. R. (2016). Carry‐over effects provide linkages across the annual cycle of a Neotropical migratory bird, the Louisiana Waterthrush *Parkesia motacilla* . Ibis, 158, 395–406. 10.1111/ibi.12344

[ece310346-bib-0052] Low, M. , & Pärt, T. (2009). Patterns of mortality for each life‐history stage in a population of the endangered New Zealand stitchbird. Journal of Animal Ecology, 78, 761–771. 10.1111/j.1365-2656.2009.01543.x 19302320

[ece310346-bib-0053] Mayfield, H. F. (1975). Suggestions for calculating nest success. The Wilson Bulletin, 87, 456–466.

[ece310346-bib-0054] Müller, M. , Spaar, R. , Schifferli, L. , & Jenni, L. (2005). Effects of changes in farming of subalpine meadows on a grassland bird, the whinchat (*Saxicola rubetra*). Journal of Ornithology, 146, 14–23. 10.1007/s10336-004-0059-0

[ece310346-bib-0055] Murray, C. , Minderman, J. , Allison, J. , & Calladine, J. (2016). Vegetation structure influences foraging decisions in a declining grassland bird: The importance of fine‐scale habitat and grazing regime. Bird Study, 63, 223–232. 10.1080/00063657.2016.1180342

[ece310346-bib-0056] Naef‐Daenzer, B. , & Grüebler, M. U. (2016). Post‐fledging survival of altricial birds: Ecological determinants and adaptation. Journal of Field Ornithology, 87, 227–250. 10.1111/jofo.12157

[ece310346-bib-0057] Newton, I. (2004). Population limitation in migrants. Ibis, 146, 197–226. 10.1111/j.1474-919X.2004.00293.x

[ece310346-bib-0058] Öberg, M. , Arlt, D. , Pärt, T. , Laugen, A. T. , Eggers, S. , & Low, M. (2015). Rainfall during parental care reduces reproductive and survival components of fitness in a passerine bird. Ecology and Evolution, 5, 345–356. 10.1002/ece3.1345 25691962 PMC4314267

[ece310346-bib-0059] Ockendon, N. , Leech, D. , & Peace‐Higgins, J. W. (2013). Climatic effects on breeding grounds are more important drivers of breeding phenology in migrant birds than carry‐over effects from wintering grounds. Biology Letters, 9, 20130669. 10.1098/rsbl.2013.0669 24196517 PMC3871353

[ece310346-bib-0061] Pearce‐Higgins, J. W. , & Grant, M. C. (2006). Relationships between bird abundance and the composition and structure of moorland vegetation. Bird Study, 53, 112–125. 10.1080/00063650609461424

[ece310346-bib-0062] Pearce‐Higgins, J. W. , Stephen, L. , Langston, R. H. , Bainbridge, I. P. , & Bullman, R. (2009). The distribution of breeding birds around upland wind farms. Journal of Applied Ecology, 46, 1323–1331. 10.1111/j.1365-2664.2009.01715.x

[ece310346-bib-0063] PECBMS . (2020). PanEuropean common bird monitoring scheme for European trends .

[ece310346-bib-0065] Pierce, D. (2021). ncdf4: Interface to Unidata netCDF (version 4 or earlier) format data files . R Package Version 1.19. https://CRAN.R‐project.org/package=ncdf4

[ece310346-bib-0066] R Core Team . (2020). R: A language and environment for statistical computing. R Foundation for Statistical Computing.

[ece310346-bib-0067] Radford, A. N. , McCleery, R. H. , Woodburn, R. J. W. , & Morecroft, M. D. (2010). Activity patterns of parent great tits *Parus major* feeding their young during rainfall. Bird Study, 48, 214–220. 10.1080/00063650109461220

[ece310346-bib-0068] Risely, A. , Blackburn, E. , & Cresswell, W. (2015). Patterns in departure phenology and mass gain on African non‐breeding territories prior to the Sahara crossing in a long‐distance migrant. Ibis, 157, 808–822. 10.1111/ibi.12288

[ece310346-bib-0070] Rushing, C. S. , Marra, P. P. , & Dudash, M. R. (2016). Winter habitat quality but not long‐distance dispersal influences apparent reproductive success in a migratory bird. Ecology, 97, 1218–1227. 10.1890/15-1259.1 27349098

[ece310346-bib-0071] Saino, N. , Bazzi, G. , Gatti, E. , Caprioli, M. , Cecere, J. G. , Possenti, C. D. , Galimberti, A. , Orioli, V. , Bani, L. , Rubolini, D. , Gianfranceschi, L. , & Spina, F. (2015). Polymorphism at the *clock* gene predicts phenology of long‐distance migration in birds. Molecular Ecology, 24, 1758–1773. 10.1111/mec.13159 25780812

[ece310346-bib-0072] Shitikov, D. , Fedotova, S. , Gagieva, V. , Fedchuk, D. , Dubkova, E. , & Vaytina, T. (2012). Breeding‐site fidelity and dispersal in isolated populations of three migratory passerines. Ornis Fennica, 89, 53–62.

[ece310346-bib-0073] Shitikov, D. A. , Vaytina, T. M. , Gagieva, V. A. , & Fedchuk, D. V. (2015). Breeding success affects site fidelity in a whinchat *Saxicola rubetra* population in abandoned fields. Bird Study, 62, 96–105. 10.1080/00063657.2014.988120

[ece310346-bib-0074] Skagen, S. K. , & Yackel Adams, A. A. (2012). Weather effects on avian breeding performance and implications of climate change. Ecological Appications., 22, 1131–1145. 10.1890/11-0291.1 22827123

[ece310346-bib-0075] Smith, R. J. , & Moore, F. R. (2003). Arrival fat and reproductive performance in a long‐distance passerine migrant. Oecologia, 134, 325–331. 10.1007/s00442-002-1152-9 12647139

[ece310346-bib-0076] Sosnovcová, K. , Koleček, J. , Požgayová, M. , Jelínek, V. , Šulc, M. , Steidlová, P. , Honza, M. , & Procházka, P. (2018). Timing of natal nests is an important factor affecting return rates of juvenile great reed warblers. Journal of Ornithology, 159, 183–190. 10.1007/s10336-017-1492-1

[ece310346-bib-0078] Stanbury, A. J. , Tománková, I. , Teuten, E. L. , & Douglas, D. J. T. (2022). No evidence that declining whinchat *Saxicola rubetra* are currently limited by the availability of apparently suitable breeding habitat within the UK uplands. Journal of Ornithology, 163, 273–283. 10.1007/s10336-021-01925-6

[ece310346-bib-0079] Tome, D. , & Denac, D. (2012). Survival and development of predator avoidance in the post‐fledging period of the whinchat (*Saxicola rubetra*): Consequences for conservation measures. Journal of Ornithology, 153, 131–138. 10.1007/s10336-011-0713

[ece310346-bib-0080] Tome, D. , Denac, D. , & Vrezec, A. (2020). Mowing is the greatest threat to whinchat *Saxicola rubetra* nests even when compared to several natural induced threats. Journal of Nature Conservation, 54, 125781. 10.1016/j.jnc.2019.125781

[ece310346-bib-0082] Verhulst, S. , & Nilsson, J.‐A. (2007). The timing of birds' breeding seasons: A review of experiments that manipulated timing of breeding. Philosophical Transactions of the Royal Society, B: Biological Sciences, 363, 399–410. 10.1098/rstb.2007.2146 PMC2606757 17666390

[ece310346-bib-0084] Vickery, J. A. , Ewing, S. R. , Smith, K. W. , Pain, D. J. , Bairlein, F. , Škorpilová, J. , & Gregory, R. D. (2014). The decline of afro‐Palaearctic migrants and an assessment of potential causes. Ibis, 156, 1–22. 10.1111/ibi.12118

[ece310346-bib-0085] Visser, M. E. , van Noordwijk, A. J. , Tinbergen, J. M. , & Lessells, C. M. (1998). Warmer springs lead to mistimed reproduction in great tits (*Parus major*). Proceedings of the Royal Society of London B: Biological Sciences, 265, 1867–1870. 10.1098/rspb.1998.0514

[ece310346-bib-0086] Vitz, A. C. , & Rodewald, A. D. (2011). Influence of condition and habitat use on survival of post‐fledging songbirds. The Condor, 113, 400–411. 10.1525/cond.2011.100023

[ece310346-bib-0087] Wickham, H. (2016). ggplot2: Elegant graphics for data analysis. Springer‐Verlag.

[ece310346-bib-0088] Wilke, C. (2020). Cowplot: Streamlined plot theme and plot annotations for 'ggplot2' . R Package Version 1.1.1. https://CRAN.R‐project.org/package=cowplot

[ece310346-bib-0090] Woodward, I. , Massimino, D. , Hammond, M. , Barber, L. , Barimore, C. , Harris, S. , Leech, D. , Noble, D. G. , Walker, R. , Baillie, S. R. , & Robinson, R. A. (2020). BirdTrends 2020: Trends in numbers, breeding success and survival for UK breeding birds . BTO Research Report 732, BTO, Thetford. http://www.bto.org/birdtrends

