# Supplementary material for: Early life conditions influence fledging success and subsequent local recruitment rates in a declining migratory songbird, the Whinchat Saxicola rubetra
Source: Ecol Evol. 2023 Jul 20;13(7):e10346. doi: 10.1002/ece3.10346 (PMC10361359; doi:10.1002/ece3.10346)
Supplement: Supplementary file 1 — Appendix S1 [file ECE3-13-e10346-s001.docx]

Supplementary material for ‘Early life conditions influence fledging success and subsequent local recruitment rates in a declining migratory songbird, the Whinchat *Saxicola rubetra*’.

Chay Halliwell, Martin Ketcher, Amanda Proud, Stephen Westerberg, David J.T. Douglas, Malcolm D. Burgess


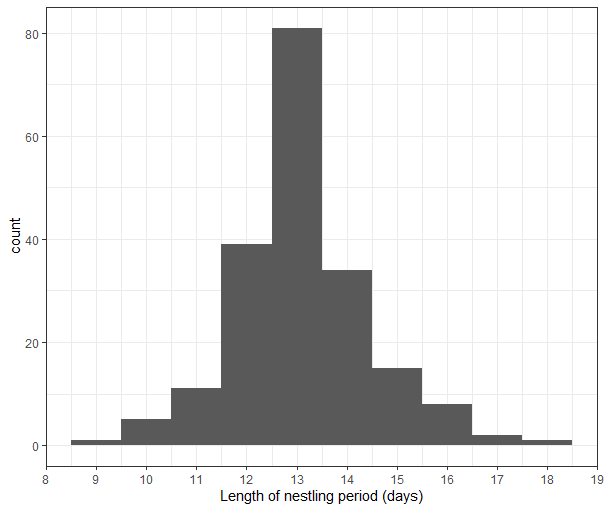


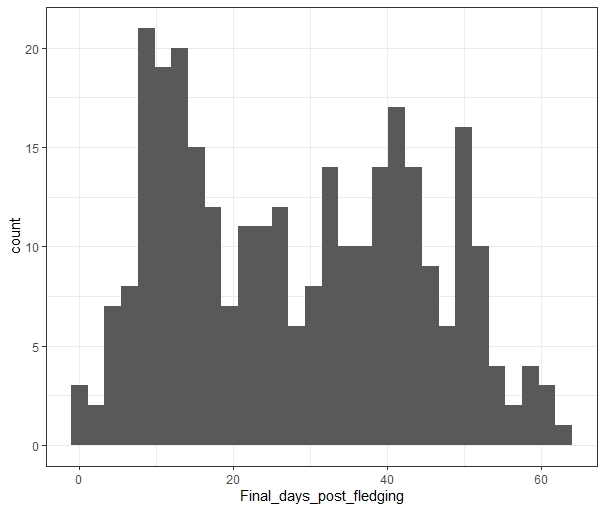


Figure S1. Histogram of number of days between hatching and fledging, median - 13 days, N = 197.

Figure S2. Histogram of frequency of final resightings of fledglings within their fledging year against the number of days post-fledging on which the resighting occurred, N = 311.

| Table S1. Medians and ranges for vegetation coverage within 5m, 50m and 200m. | | | | | | | |
| --- | --- | --- | --- | --- | --- | --- | --- |
| **Vegetation type** | | **5m** | | **50m** | | **200m** | |
| **Common name** | **Latin genus** | **Mean** | **Range** | **Mean** | **Range** | **Mean** | **Range** |
| Bracken | *Pteridium* | 43.5 | 0-100 | 46.6 | 0-95 | 42.7 | 0-90 |
| Tree scrub | *e.g. Crataegus* | 0.3 | 0-10 | 0.8 | 0-15 | 2.0 | 0-15 |
| Tufted hair grass | *Deschampsia* | 2.1 | 0-50 | 1.9 | 0-30 | 1.1 | 0-15 |
| Purple moor grass | *Molinia* | 8.4 | 0-100 | 6.1 | 0-65 | 7.3 | 0-65 |
| Rush | *Juncus* | 6.5 | 0-79 | 13.5 | 0-70 | 16.0 | 0-60 |
| Bilberry | *Vaccinium* | 2.4 | 0-45 | 1.6 | 0-30 | 1.5 | 0-25 |
| Heather | *Calluna* | 3.7 | 0-90 | 3.0 | 0-50 | 5.7 | 0-35 |
| Other grass | *e.g.* *Holcus* | 33.1 | 0-100 | 26.6 | 0-96 | 23.4 | 0-86 |

| Table S2. Single term (and two term) model outputs for variables influencing probability of a nest surviving to fledging from the vegetation sampled data subset, including both nests which fledged, and which did not. Used to determine terms for subsequent inclusion in the Binomially distributed GLMM ‘Nest success vegetation years’. Terms with P ≤ 0.1 in bold. N = 143. | | | | |
| --- | --- | --- | --- | --- |
| **Variable** | **χ2** | **P-value** | **Included in model?** |  |
| Year | 5.2 | 0.27 | Yes, as random effect |  |
| Altitude | 0.99 | 0.32 | No |  |
| Altitude^2^ | 0.098 | 0.75 | No |  |
| Lay date | 1.1 | 0.29 | No |  |
| Lay date^2^ | 0.42 | 0.52 | No |  |
| Hatch date | 0.50 | 0.48 | No |  |
| Hatch date^2^ | 0.90 | 0.34 | No |  |
| **Clutch size** | **7.6** | **0.0060** | **No, correlation with brood size** |  |
| Clutch size^2^ | 0.089 | 0.77 | No |  |
| **Brood size** | **11.6** | **0.00066** | **Yes** |  |
| Brood size^2^ | 0.35 | 0.56 | No |  |
| Nest substrate | 2.4 | 0.87 | No |  |
| Bracken 5m | 1.1 | 0.30 | No |  |
| **(Bracken 5m) ^2^** | **3.3** | **0.068** | **No, correlation with bracken 50m** |  |
| Bracken 50m | 0.54 | 0.46 | Yes |  |
| **(Bracken 50m) ^2^** | **4.5** | **0.033** | **Yes** |  |
| Bracken 200m | 0.97 | 0.33 | No |  |
| (Bracken 200m) ^2^ | 2.3 | 0.13 | No |  |
| **Tree scrub 5m** | **2.9** | **0.088** | **Yes** |  |
| (Tree scrub 5m) ^2^ | 0.19 | 0.66 | No |  |
| Tree scrub 50m | 2.1 | 0.15 | No |  |
| (Tree scrub 50m) ^2^ | 0.95 | 0.33 | No |  |
| **Tree scrub 200m** | **3.6** | **0.059** | **Yes** |  |
| (Tree scrub 200m) ^2^ | 0.066 | 0.80 | No |  |
| Tufted hair grass 5m | 1.7 | 0.20 | No |  |
| (Tufted hair grass 5m) ^2^ | 0.68 | 0.41 | No |  |
| Tufted hair grass 50m | 0.86 | 0.35 | No |  |
| (Tufted hair grass 50m) ^2^ | 0.38 | 0.54 | No |  |
| Tufted hair grass 200m | 1.1 | 0.29 | No |  |
| (Tufted hair grass 200m) ^2^ | 0.78 | 0.38 | No |  |
| Purple moor grass 5m | 0.73 | 0.39 | No |  |
| (Purple moor grass 5m) ^2^ | 1.9 | 0.17 | No |  |
| Purple moor grass 50m | 0.94 | 0.33 | No |  |
| (Purple moor grass 50m) ^2^ | 0.34 | 0.56 | No |  |
| Purple moor grass 200m | 0.15 | 0.70 | No |  |
| (Purple moor grass 200m) ^2^ | 0.89 | 0.34 | No |  |
| Other grass 5m | 1.5 | 0.22 | No |  |
| (Other grass 5m) ^2^ | 1.3 | 0.26 | No |  |
| Other grass 50m | 0.13 | 0.72 | No |  |
| (Other grass 50m) ^2^ | 1.8 | 0.17 | No |  |
| Other grass 200m | 0.089 | 0.77 | No |  |
| (Other grass 200m) ^2^ | 0.24 | 0.63 | No |  |
| Rush 5m | <0.001 | 0.99 | No |  |
| (Rush 5m) ^2^ | 0.21 | 0.65 | No |  |
| Rush 50m | 1.1 | 0.30 | No |  |
| (Rush 50m) ^2^ | 0.48 | 0.49 | No |  |
| Rush 200m | 2.3 | 0.13 | No |  |
| (Rush 200m) ^2^ | 0.056 | 0.81 | No |  |
| Bilberry 5m | 0.19 | 0.66 | No |  |
| (Bilberry 5m) ^2^ | 1.4 | 0.24 | No |  |
| Bilberry 50m | 0.72 | 0.40 | No |  |
| (Bilberry 50m) ^2^ | 0.60 | 0.44 | No |  |
| Bilberry 200m | 0.044 | 0.83 | No |  |
| (Bilberry 200m) ^2^ | 2.5 | 0.12 | No |  |
| Heather 5m | 1.7 | 0.19 | No |  |
| (Heather 5m) ^2^ | 1.3 | 0.25 | No |  |
| Heather 50m | 0.063 | 0.80 | Yes |  |
| **(Heather 50m) ^2^** | **3.0** | **0.085** | **Yes** |  |
| Heather 200m | 0.45 | 0.50 | No |  |
| (Heather 200m) ^2^ | 1.2 | 0.27 | No |  |
| No. trees 5m | 0.89 | 0.35 | No |  |
| (No. trees 5m) ^2^ | 0.31 | 0.58 | No |  |
| No. trees 50m | 1.6 | 0.90 | No |  |
| No. trees 200m | 6.7 | 0.75 | No |  |
| **Ditch/stream 5m** | **2.8** | **0.093** | **Yes** |  |
| Ditch/stream 50m | 1.1 | 0.30 | No |  |
| Ditch/stream 200m | 0.35 | 0.56 | No |  |
| Fence posts 5m | <0.001 | 0.99 | No |  |
| Fence posts 50m | 0.12 | 0.73 | No |  |
| Fence posts 200m | 0.17 | 0.68 | No |  |
| Overhead wires 5m | <0.001 | 0.98 | No |  |
| Overhead wires 50m | 0.70 | 0.40 | No |  |
| **Overhead wires 200m** | **4.2** | **0.039** | **Yes** |  |
| Path 5m | 1.4 | 0.23 | No |  |
| **Path 50m** | **2.8** | **0.093** | **Yes** |  |
| Path 200m | 0.0014 | 0.97 | No |  |
| Boulders 5m | 0.0017 | 0.97 | No |  |
| Boulders 50m | 0.0067 | 0.93 | No |  |
| Boulders 200m | 2.1 | 0.14 | No |  |
| Wall 5m | <0.001 | 0.99 | No |  |
| Wall 50m | 1.4 | 0.24 | No |  |
| Wall 200m | 1.4 | 0.24 | No |  |
| Mean temp. in-nest | 1.8 | 0.18 | No |  |
| **Mean temp. in-nest^2^** | **3.2** | **0.074** | **Yes** |  |
| Total rainfall in-nest | 1.1 | 0.29 | No |  |
| Total rainfall in-nest^2^ | 0.13 | 0.72 | No |  |

| Table S3. Single term (and two term) model outputs for variables influencing probability of at least one fledgling being recruited into the local population from the vegetation sampled data subset but including only nests which fledged. Used to determine terms for subsequent inclusion in the Binomially distributed GLMM ‘Local recruitment vegetation years’. Terms with P ≤ 0.1 in bold. N = 118. | | | | |
| --- | --- | --- | --- | --- |
| **Variable** | **χ2** | **P-value** | **Included in model?** |  |
| Year | 4.6 | 0.33 | Yes, as random effect |  |
| Altitude | 0.26 | 0.61 | Yes |  |
| **Altitude^2^** | **3.5** | **0.060** | **Yes** |  |
| **Lay date** | **9.0** | **0.0027** | Yes |  |
| Lay date^2^ | 0.079 | 0.78 | No |  |
| **Hatch date** | **8.0** | **0.0047** | **No, correlation with lay date** |  |
| Hatch date^2^ | 0.22 | 0.64 | No |  |
| **Clutch size** | **8.27** | **0.0040** | **No, correlation with brood size** |  |
| Clutch size^2^ | 1.5 | 0.22 | No |  |
| **Brood size** | **10.2** | **0.0014** | **Yes** |  |
| Brood size^2^ | 1.1 | 0.31 | No |  |
| Nest substrate | 0.83 | 0.99 | No |  |
| Bracken 5m | 0.67 | 0.41 | No |  |
| (Bracken 5m) ^2^ | 1.6 | 0.20 | No |  |
| Bracken 50m | 1.6 | 0.20 | No |  |
| (Bracken 50m) ^2^ | 0.16 | 0.69 | No |  |
| **Bracken 200m** | **3.8** | **0.051** | **Yes** |  |
| (Bracken 200m) ^2^ | 0.087 | 0.77 | No |  |
| Tree scrub 5m | 1.5 | 0.22 | No |  |
| (Tree scrub 5m) ^2^ | 1.2 | 0.28 | No |  |
| Tree scrub 50m | 0.12 | 0.73 | No |  |
| (Tree scrub 50m) ^2^ | 0.47 | 0.49 | No |  |
| Tree scrub 200m | 0.40 | 0.53 | No |  |
| (Tree scrub 200m) ^2^ | 2.5 | 0.11 | No |  |
| Tufted hair grass 5m | 1.3 | 0.25 | No |  |
| (Tufted hair grass 5m) ^2^ | 0.58 | 0.45 | No |  |
| Tufted hair grass 50m | 2.6 | 0.11 | No |  |
| (Tufted hair grass 50m) ^2^ | 0.72 | 0.40 | No |  |
| Tufted hair grass 200m | 2.6 | 0.10 | No |  |
| (Tufted hair grass 200m) ^2^ | 0.19 | 0.66 | No |  |
| Purple moor grass 5m | 1.6 | 0.20 | No |  |
| (Purple moor grass 5m) ^2^ | 1.9 | 0.16 | No |  |
| Purple moor grass 50m | 0.16 | 0.69 | No |  |
| (Purple moor grass 50m) ^2^ | 0.13 | 0.72 | No |  |
| Purple moor grass 200m | 0.77 | 0.38 | No |  |
| (Purple moor grass 200m) ^2^ | 0.36 | 0.55 | No |  |
| Other grass 5m | 0.0044 | 0.95 | No |  |
| (Other grass 5m) ^2^ | 0.63 | 0.43 | No |  |
| Other grass 50m | 0.69 | 0.41 | No |  |
| (Other grass 50m) ^2^ | 0.29 | 0.59 | No |  |
| Other grass 200m | 0.66 | 0.42 | No |  |
| (Other grass 200m) ^2^ | 0.40 | 0.53 | No |  |
| Rush 5m | 2.3 | 0.13 | No |  |
| (Rush 5m) ^2^ | 0.0047 | 0.95 | No |  |
| **Rush 50m** | **4.9** | **0.027** | **Yes** |  |
| (Rush 50m) ^2^ | 0.20 | 0.66 | No |  |
| **Rush 200m** | **4.7** | **0.031** | **Yes** |  |
| (Rush 200m) ^2^ | 1.4 | 0.23 | No |  |
| **Bilberry 5m** | **3.8** | **0.051** | **Yes** |  |
| (Bilberry 5m) ^2^ | 0.047 | 0.83 | No |  |
| Bilberry 50m | 1.5 | 0.22 | No |  |
| (Bilberry 50m) ^2^ | 0.82 | 0.36 | No |  |
| Bilberry 200m | 0.15 | 0.70 | No |  |
| (Bilberry 200m) ^2^ | 0.045 | 0.83 | No |  |
| Heather 5m | 1.3 | 0.25 | No |  |
| (Heather 5m) ^2^ | 1.7 | 0.19 | No |  |
| **Heather 50m** | **3.5** | **0.061** | **Yes** |  |
| (Heather 50m) ^2^ | 1.8 | 0.18 | No |  |
| **Heather 200m** | **4.7** | **0.030** | **Yes** |  |
| (Heather 200m) ^2^ | 0.0060 | 0.94 | No |  |
| No. trees 5m | 1.1 | 0.30 | No |  |
| (No. trees 5m) ^2^ | 0.42 | 0.52 | No |  |
| No. trees 50m | 4.1 | 0.54 | No |  |
| No. trees 200m | 2.9 | 0.98 | No |  |
| Ditch/stream 5m | <0.001 | 1.00 | No |  |
| Ditch/stream 50m | 0.02 | 0.88 | No |  |
| Ditch/stream 200m | 0.44 | 0.51 | No |  |
| Fence posts 5m | <0.001 | 1.00 | No |  |
| Fence posts 50m | 1.6 | 0.20 | No |  |
| Fence posts 200m | 0.078 | 0.78 | No |  |
| Overhead wires 5m | <0.001 | 0.99 | No |  |
| Overhead wires 50m | 0.23 | 0.63 | No |  |
| Overhead wires 200m | 1.3 | 0.26 | No |  |
| **Path 5m** | **2.8** | **0.093** | **Yes** |  |
| Path 50m | 2.4 | 0.12 | No |  |
| Path 200m | 0.43 | 0.51 | No |  |
| Boulders 5m | 1.4 | 0.23 | No |  |
| Boulders 50m | 0.18 | 0.67 | No |  |
| Boulders 200m | 0.0045 | 0.95 | No |  |
| Wall 5m | 0.22 | 0.64 | No |  |
| Wall 50m | 0.46 | 0.50 | No |  |
| Wall 200m | 0.53 | 0.47 | No |  |
| **Mean temp. in-nest** | **7.1** | **0.0076** | **Yes** |  |
| Mean temp. in-nest^2^ | 1.2 | 0.28 | No |  |
| Total rainfall in-nest | 1.5 | 0.23 | No |  |
| Total rainfall in-nest^2^ | 2.3 | 0.13 | No |  |
| **Mean temp. 50d post-fledge** | **4.8** | **0.028** | **Yes** |  |
| Mean temp. 50d post-fledge^2^ | 0.24 | 0.62 | No |  |
| **Mean temp. 40d post-fledge** | **3.2** | **0.072** | **No, correlation with mean temp 50d** |  |
| Mean temp. 40d post-fledge^2^ | 0.075 | 0.78 | No |  |
| Total rainfall 50d post-fledge | 0.18 | 0.70 | No |  |
| Total rainfall 50d post-fledge^2^ | 0.27 | 0.61 | No |  |
| Total rainfall 40d post-fledge | 0.75 | 0.39 | No |  |
| Total rainfall 40d post-fledge^2^ | 0.32 | 0.57 | No |  |

| Table S4. Single term (and two term) model outputs for variables influencing the proportion of fledglings which were recruited into the local population from the vegetation sampled data subset but including only nests which fledged. Used to determine terms for subsequent inclusion in the Binomially distributed GLMM ‘Proportion recruited vegetation years’. Terms with P ≤ 0.1 in bold. N = 118. | | | | |
| --- | --- | --- | --- | --- |
| Variable | χ2 | P-value | Included in model? |  |
| Year | 6.0 | 0.20 | Yes, as random effect |  |
| Altitude | 0.60 | 0.44 | No |  |
| Altitude^2^ | 0.32 | 0.57 | No |  |
| Lay date | 2.2 | 0.14 | No |  |
| Lay date^2^ | 1.7 | 0.19 | No |  |
| Hatch date | 2.0 | 0.16 | No |  |
| Hatch date^2^ | 1.9 | 0.17 | No |  |
| Clutch size | 2.7 | 0.10 | No, correlation with brood size |  |
| Clutch size^2^ | 2.2 | 0.14 | No |  |
| **Brood size** | **5.5** | **0.02** | **Yes** |  |
| Brood size^2^ | 0.81 | 0.37 | No |  |
| Nest substrate | 4.0 | 0.67 | No |  |
| Bracken 5m | 0.86 | 0.35 | No |  |
| (Bracken 5m) ^2^ | 0.93 | 0.33 | No |  |
| Bracken 50m | 0.049 | 0.83 | No |  |
| (Bracken 50m) ^2^ | 0.11 | 0.74 | No |  |
| Bracken 200m | 0.26 | 0.61 | No |  |
| (Bracken 200m) ^2^ | 1.1 | 0.30 | No |  |
| Tree scrub 5m | 1.9 | 0.17 | No |  |
| (Tree scrub 5m) ^2^ | 0.35 | 0.55 | No |  |
| Tree scrub 50m | 2.1 | 0.15 | No |  |
| (Tree scrub 50m) ^2^ | 1.5 | 0.23 | No |  |
| **Tree scrub 200m** | **0.15** | **0.70** | **Yes** |  |
| **(Tree scrub 200m) ^2^** | **4.86** | **0.028** | **Yes** |  |
| Tufted hair grass 5m | 0.24 | 0.62 | No |  |
| (Tufted hair grass 5m) ^2^ | 2.3 | 0.13 | No |  |
| Tufted hair grass 50m | 0.53 | 0.47 | No |  |
| (Tufted hair grass 50m) ^2^ | 2.39 | 0.12 | No |  |
| Tufted hair grass 200m | 0.29 | 0.59 | No |  |
| (Tufted hair grass 200m) ^2^ | 2.3 | 0.13 | No |  |
| Purple moor grass 5m | 2.7 | 0.10 | No |  |
| (Purple moor grass 5m) ^2^ | 1.9 | 0.16 | No |  |
| Purple moor grass 50m | 0.41 | 0.52 | No |  |
| (Purple moor grass 50m) ^2^ | 0.69 | 0.41 | No |  |
| Purple moor grass 200m | 1.6 | 0.20 | No |  |
| (Purple moor grass 200m) ^2^ | 0.82 | 0.37 | No |  |
| Other grass 5m | 0.29 | 0.59 | No |  |
| (Other grass 5m) ^2^ | 0.13 | 0.72 | No |  |
| Other grass 50m | 0.78 | 0.38 | No |  |
| (Other grass 50m) ^2^ | 2.3 | 0.13 | No |  |
| **Other grass 200m** | **3.1** | **0.08** | **Yes** |  |
| (Other grass 200m) ^2^ | 0.19 | 0.66 | No |  |
| Rush 5m | 0.088 | 0.77 | No |  |
| (Rush 5m) ^2^ | 0.021 | 0.89 | No |  |
| Rush 50m | 2.28 | 0.13 | No |  |
| (Rush 50m) ^2^ | 0.69 | 0.41 | No |  |
| **Rush 200m** | **2.7** | **0.10** | **Yes** |  |
| (Rush 200m) ^2^ | 1.5 | 0.22 | No |  |
| Bilberry 5m | 2.4 | 0.12 | No |  |
| (Bilberry 5m) ^2^ | 0.018 | 0.89 | No |  |
| Bilberry 50m | 0.88 | 0.35 | No |  |
| (Bilberry 50m) ^2^ | 1.72 | 0.19 | No |  |
| Bilberry 200m | 0.65 | 0.42 | No |  |
| (Bilberry 200m) ^2^ | 0.12 | 0.73 | No |  |
| Heather 5m | 0.00 | 1.0 | No |  |
| (Heather 5m) ^2^ | 0.68 | 0.41 | No |  |
| **Heather 50m** | **1.84** | **0.17** | **Yes** |  |
| **(Heather 50m) ^2^** | **4.3** | **0.037** | **Yes** |  |
| Heather 200m | 0.37 | 0.54 | No |  |
| (Heather 200m) ^2^ | 0.11 | 0.74 | No |  |
| No. trees 5m | 1.9 | 0.16 | No |  |
| (No. trees 5m) ^2^ | 0.64 | 0.42 | No |  |
| No. trees 50m | 2.76 | 0.74 | No |  |
| No. trees 200m | 9.97 | 0.44 | No |  |
| Ditch/stream 5m | 0.00 | 0.99 | No |  |
| Ditch/stream 50m | 1.94 | 0.16 | No |  |
| Ditch/stream 200m | 0.18 | 0.67 | No |  |
| **Fence posts 5m** | **4.9** | **0.027** | **Yes** |  |
| Fence posts 50m | 1.5 | 0.22 | No |  |
| Fence posts 200m | 0.43 | 0.51 | No |  |
| Overhead wires 5m | 0.00 | 0.98 | No |  |
| Overhead wires 50m | 0.065 | 0.80 | No |  |
| Overhead wires 200m | 1.2 | 0.27 | No |  |
| Path 5m | 2.3 | 0.13 | No |  |
| Path 50m | 2.4 | 0.12 | No |  |
| **Path 200m** | **6.3** | **0.012** | **Yes** |  |
| Boulders 5m | 1.1 | 0.29 | No |  |
| Boulders 50m | 0.0038 | 0.95 | No |  |
| Boulders 200m | 0.040 | 0.84 | No |  |
| Wall 5m | 1.3 | 0.25 | No |  |
| Wall 50m | 2.4 | 0.12 | No |  |
| Wall 200m | 0.56 | 0.45 | No |  |
| Mean temp. in-nest | 0.91 | 0.34 | No |  |
| Mean temp. in-nest^2^ | 2.3 | 0.13 | No |  |
| Total rainfall in-nest | 6.1 | 0.013 | No |  |
| Total rainfall in-nest^2^ | 5.7 | 0.017 | No |  |
| **Mean temp. 50d post-fledge** | **4.0** | **0.045** | **Yes** |  |
| Mean temp. 50d post-fledge^2^ | 0.27 | 0.61 | No |  |
| Mean temp. 40d post-fledge | 3.8 | 0.052 | No, correlation with 50d |  |
| Mean temp. 40d post-fledge^2^ | 0.30 | 0.58 | No |  |
| Total rainfall 50d post-fledge | 0.72 | 0.40 | No |  |
| Total rainfall 50d post-fledge^2^ | 0.65 | 0.42 | No |  |
| Total rainfall 40d post-fledge | 0.22 | 0.64 | No |  |
| Total rainfall 40d post-fledge^2^ | 1.5 | 0.23 | No |  |

| Table S5. Spearman rank correlation coefficient matrix of all continuous and integer variables from the vegetation sampled data subset, including nests which fledged and nests which did not fledge. Utilised for analysis of ‘Nest success vegetation years’ model. \|r_s_\| > 0.7 in bold. N = 143. | | | | | | | | | | | | | | | | | | | | | | | | | | | | | | | | | |  |
| --- | --- | --- | --- | --- | --- | --- | --- | --- | --- | --- | --- | --- | --- | --- | --- | --- | --- | --- | --- | --- | --- | --- | --- | --- | --- | --- | --- | --- | --- | --- | --- | --- | --- | --- |
|  | Altitude | Lay date | Hatch date | Clutch size | Brood size | Slope | Bracken 5m | Bracken 50m | Bracken 200m | Tree scrub 5m | Tree scrub 50m | Tree scrub 200m | Tufted hair grass 5m | Tufted hair grass 50m | Tufted hair grass 200m | Purple moor grass 5m | Purple moor grass 50m | Purple moor grass 200m | Other grass 5m | Other grass 50m | Other grass 200m | Rush 5m | Rush 50m | Rush 200m | Bilberry 5m | Bilberry 50m | Bilberry 200m | Heather 5m | Heather 50m | Heather 200m | No. trees 5m | Mean temp. in-nest | Total rainfall in-nest | |
| Total rainfall (in-nest) | .12 | -.02 | -.01 | .00 | -.04 | -.01 | .15 | .06 | .10 | -.05 | -.09 | -.14 | .04 | -.06 | -.12 | -.02 | .15 | .10 | .01 | .01 | -.11 | -.10 | -.15 | -.07 | -.05 | -.07 | -.01 | .01 | -.06 | -.08 | -.10 | -.26 |  | |
| Mean temp. (in-nest) | .03 | .40 | .40 | -.24 | -.25 | -.04 | -.04 | -.06 | -.08 | -.01 | .03 | .05 | -.10 | -.02 | -.02 | -.01 | -.01 | .07 | -.03 | -.08 | -.03 | .18 | .23 | .11 | -.03 | .09 | .07 | .01 | -.04 | .03 | -.03 |  |  |  |
| No. trees 5m | -.38 | -.11 | -.12 | .18 | .06 | -.15 | -.51 | -.52 | -.53 | .58 | .48 | .41 | .35 | .37 | .32 | .02 | -.03 | .08 | .29 | .38 | .42 | .26 | .25 | .25 | .17 | .28 | .29 | -.19 | -.19 | -.20 |  |  |  |  |
| Heather 200m | .17 | .05 | .07 | .05 | .04 | -.07 | -.03 | .01 | -.15 | -.16 | -.26 | -.19 | -.14 | -.20 | .32 | .07 | .09 | .04 | -.04 | -.08 | -.15 | -.06 | .12 | .12 | .22 | .02 | -.08 | .39 | .50 |  |  |  |  |  |
| Heather 50m | -.01 | .12 | .11 | -.06 | -.02 | -.16 | -.20 | -.22 | -.23 | -.14 | -.08 | -.04 | -.05 | -.04 | .01 | .21 | .26 | .15 | -.01 | -.01 | -.09 | -.02 | .13 | .07 | .33 | .03 | -.05 | **.72** |  |  |  |  |  |  |
| Heather 5m | -.02 | .07 | .16 | -.10 | -.07 | -.28 | -.31 | -.26 | -.22 | -.18 | -.03 | .05 | -.04 | -.03 | .07 | .26 | .28 | .20 | -.08 | -.03 | -.10 | .05 | .13 | .08 | .43 | .15 | -.04 |  |  |  |  |  |  |  |
| Bilberry 200m | .00 | .00 | .00 | .02 | .05 | -.02 | -.15 | -.24 | -.26 | .11 | .08 | -.02 | .19 | .17 | .11 | -.07 | -.07 | .03 | .11 | .15 | .02 | .17 | .09 | .19 | .19 | .45 |  |  |  |  |  |  |  |  |
| Bilberry 50m | -.08 | -.01 | .05 | .12 | .06 | -.12 | -.26 | -.29 | -.31 | .02 | .20 | .10 | .04 | .12 | .15 | .05 | .05 | .11 | .16 | .15 | .16 | .21 | .10 | .20 | .47 |  |  |  |  |  |  |  |  |  |
| Bilberry 5m | -.03 | .08 | .05 | .07 | .05 | -.10 | -.28 | -.22 | -.29 | .02 | .13 | .00 | -.01 | .07 | .11 | -.05 | .04 | .08 | .11 | .08 | .10 | .13 | .09 | .17 |  |  |  |  |  |  |  |  |  |  |
| Rush 200m | -.12 | .10 | .09 | .09 | -.02 | -.16 | -.35 | -.45 | -.57 | .12 | -.01 | .08 | .15 | .25 | .20 | .16 | .10 | .15 | .08 | .18 | .08 | .34 | .52 |  |  |  |  |  |  |  |  |  |  |  |
| Rush 50m | -.15 | .19 | .16 | -.03 | -.10 | -.09 | -.36 | -.46 | -.42 | .13 | .10 | .18 | .16 | .19 | .13 | .11 | .11 | .13 | .05 | .00 | .07 | .57 |  |  |  |  |  |  |  |  |  |  |  |  |
| Rush 5m | -.03 | .04 | .01 | .00 | -.08 | -.01 | -.32 | -.37 | -.31 | .16 | .14 | .20 | .29 | .33 | .25 | -.12 | -.08 | .00 | .16 | .15 | .12 |  |  |  |  |  |  |  |  |  |  |  |  |  |
| Other grass 200m | -.15 | .05 | .07 | .10 | .07 | .01 | -.46 | -.45 | -.53 | .24 | .29 | .19 | .27 | .34 | .29 | -.11 | -.27 | -.24 | .51 | .68 |  |  |  |  |  |  |  |  |  |  |  |  |  |  |
| Other grass 50m | -.13 | -.06 | -.03 | .15 | .15 | -.04 | -.60 | -.66 | -.50 | .27 | .32 | .17 | .30 | .39 | .38 | -.13 | -.25 | -.18 | **.70** |  |  |  |  |  |  |  |  |  |  |  |  |  |  |  |
| Other grass 5m | -.08 | .04 | .04 | .00 | -.03 | .01 | -.57 | -.42 | -.34 | .17 | .19 | .15 | .31 | .36 | .30 | -.41 | -.33 | -.20 |  |  |  |  |  |  |  |  |  |  |  |  |  |  |  |  |
| Purple moor grass 200m | -.10 | .04 | .06 | .02 | .02 | -.28 | -.16 | -.25 | -.34 | -.01 | -.02 | -.03 | -.12 | .20 | -.08 | .46 | **.71** |  |  |  |  |  |  |  |  |  |  |  |  |  |  |  |  |  |
| Purple moor grass 50m | -.12 | .08 | .12 | -.06 | -.07 | -.28 | -.14 | -.25 | -.21 | -.11 | -.11 | -.09 | -.19 | -.27 | -.19 | .69 |  |  |  |  |  |  |  |  |  |  |  |  |  |  |  |  |  |  |
| Purple moor grass 5m | -.18 | .00 | .06 | .02 | .08 | -.28 | -.23 | -.30 | -.23 | .01 | -.01 | .02 | -.15 | -.15 | -.02 |  |  |  |  |  |  |  |  |  |  |  |  |  |  |  |  |  |  |  |
| Tufted hair grass 200m | -.24 | -.10 | -.09 | .14 | .04 | -.12 | -.43 | -.42 | -.35 | .45 | .42 | .40 | .59 | **.76** |  |  |  |  |  |  |  |  |  |  |  |  |  |  |  |  |  |  |  |  |
| Tufted hair grass 50m | -.22 | -.09 | -.09 | .07 | -.01 | .01 | -.40 | -.40 | -.31 | .40 | .34 | .32 | **.77** |  |  |  |  |  |  |  |  |  |  |  |  |  |  |  |  |  |  |  |  |  |
| Tufted hair grass 5m | -.16 | -.13 | -.13 | .06 | .01 | .05 | -.32 | -.34 | -.27 | .38 | .26 | .23 |  |  |  |  |  |  |  |  |  |  |  |  |  |  |  |  |  |  |  |  |  |  |
| Tree scrub 200m | -.40 | -.26 | -.26 | .09 | -.02 | -.17 | -.36 | -.32 | -.29 | .54 | .69 |  |  |  |  |  |  |  |  |  |  |  |  |  |  |  |  |  |  |  |  |  |  |  |
| Tree scrub 50m | -.39 | -.19 | -.19 | .20 | .13 | -.13 | -.35 | -.41 | -.30 | .56 |  |  |  |  |  |  |  |  |  |  |  |  |  |  |  |  |  |  |  |  |  |  |  |  |
| Tree scrub 5m | -.28 | -.19 | -.20 | .14 | .04 | -.16 | -.31 | -.31 | -.29 |  |  |  |  |  |  |  |  |  |  |  |  |  |  |  |  |  |  |  |  |  |  |  |  |  |
| Bracken 200m | .33 | -.10 | -.13 | -.16 | -.08 | .29 | **.71** | **.81** |  |  |  |  |  |  |  |  |  |  |  |  |  |  |  |  |  |  |  |  |  |  |  |  |  |  |
| Bracken 50m | .38 | -.05 | -.08 | -.10 | -.03 | .31 | **.86** |  |  |  |  |  |  |  |  |  |  |  |  |  |  |  |  |  |  |  |  |  |  |  |  |  |  |  |
| Bracken 5m | .36 | .00 | -.05 | -.07 | .01 | .33 |  |  |  |  |  |  |  |  |  |  |  |  |  |  |  |  |  |  |  |  |  |  |  |  |  |  |  |  |
| Slope | .33 | .22 | -.18 | -.11 | -.07 |  |  |  |  |  |  |  |  |  |  |  |  |  |  |  |  |  |  |  |  |  |  |  |  |  |  |  |  |  |
| Brood size | -.12 | -.35 | -.30 | **.82** |  |  |  |  |  |  |  |  |  |  |  |  |  |  |  |  |  |  |  |  |  |  |  |  |  |  |  |  |  |  |
| Clutch size | -.22 | .36 | -.30 |  |  |  |  |  |  |  |  |  |  |  |  |  |  |  |  |  |  |  |  |  |  |  |  |  |  |  |  |  |  |  |
| Hatch date | .03 | **.97** |  |  |  |  |  |  |  |  |  |  |  |  |  |  |  |  |  |  |  |  |  |  |  |  |  |  |  |  |  |  |  |  |
| Lay date | .04 |  |  |  |  |  |  |  |  |  |  |  |  |  |  |  |  |  |  |  |  |  |  |  |  |  |  |  |  |  |  |  |  |  |
| Altitude |  |  |  |  |  |  |  |  |  |  |  |  |  |  |  |  |  |  |  |  |  |  |  |  |  |  |  |  |  |  |  |  |  |  |

| Table S6. Spearman rank correlation coefficient matrix of select (P ≤ 0.1 during single term analysis) continuous and integer variables from resighting data, including all nests which fledged. Utilised for analysis of models; ‘Local recruitment vegetation years’ & ‘Proportion recruited vegetation years’. \|r_s_\| > 0.7 in bold. N = 118. | | | | | | | | | | | | | | | | | | |
| --- | --- | --- | --- | --- | --- | --- | --- | --- | --- | --- | --- | --- | --- | --- | --- | --- | --- | --- |
|  | Altitude | Lay date | Hatch date | Clutch size | Brood size | Slope | Bracken 200m | Other grass 200m | Rush 50m | Rush 200m | Bilberry 5m | Heather 50m | Heather 200m | Mean temp. 40d post-fledge | Mean temp. 50d post-fledge | Mean temp. in-nest | Total rainfall in-nest |  |
| Total rainfall in-nest | .15 | -.06 | -.07 | -.04 | -.05 | -.03 | .10 | -.12 | -.19 | -.09 | -.05 | -.07 | -.05 | -.27 | -.19 | -.31 |  |  |
| Mean temp. in-nest | -.05 | .40 | .38 | -.23 | -.27 | -.05 | -.12 | .01 | .30 | .10 | .00 | .01 | .05 | -.53 | -.64 |  |  |  |
| Mean temp. 50d post-fledge | -.11 | -.10 | -.06 | .20 | .22 | -.02 | -.01 | .21 | -.19 | -.02 | .00 | .04 | -.04 | **.93** |  |  |  |  |
| Mean temp. 40d post-fledge | -.10 | .10 | .14 | .07 | .10 | .06 | .00 | .20 | -.14 | .03 | .04 | .06 | -.03 |  |  |  |  |  |
| Heather 200m | .19 | .06 | .05 | .04 | .02 | -.05 | -.18 | -.12 | .14 | .20 | .26 | .51 |  |  |  |  |  |  |
| Heather 50m | .08 | .11 | .12 | -.12 | -.08 | -.14 | -.23 | -.15 | .15 | .11 | .35 |  |  |  |  |  |  |  |
| Bilberry 5m | .00 | .08 | .07 | -.01 | .02 | -.13 | -.31 | .13 | .11 | .13 |  |  |  |  |  |  |  |  |
| Rush 200m | -.05 | .13 | .12 | -.01 | -.07 | -.14 | -.55 | .05 | .53 |  |  |  |  |  |  |  |  |  |
| Rush 50m | -.11 | .27 | .26 | -.04 | -.12 | -.04 | -.40 | .07 |  |  |  |  |  |  |  |  |  |  |
| Other grass 200m | -.08 | .08 | .10 | .07 | .08 | .03 | -.52 |  |  |  |  |  |  |  |  |  |  |  |
| Bracken 200m | .25 | -.18 | -.20 | -.08 | -.04 | .22 |  |  |  |  |  |  |  |  |  |  |  |  |
| Slope | .29 | .21 | .21 | -.13 | -.12 |  |  |  |  |  |  |  |  |  |  |  |  |  |
| Brood size | -.08 | -.42 | -.36 | **.84** |  |  |  |  |  |  |  |  |  |  |  |  |  |  |
| Clutch size | -.18 | -.42 | -.35 |  |  |  |  |  |  |  |  |  |  |  |  |  |  |  |
| Hatch date | -.05 | **.99** |  |  |  |  |  |  |  |  |  |  |  |  |  |  |  |  |
| Lay date | -.02 |  |  |  |  |  |  |  |  |  |  |  |  |  |  |  |  |  |
| Altitude |  |  |  |  |  |  |  |  |  |  |  |  |  |  |  |  |  |  |

| Table S7. Single term (and two term) model outputs for variables influencing probability of a nest surviving to fledging from the full dataset, including both nests which fledged, and which did not. Used to determine terms for subsequent inclusion in the Binomially distributed GLMM ‘Nest success all years’. Terms with P ≤ 0.1 in bold. N = 247. | | | | |
| --- | --- | --- | --- | --- |
| **Variable** | **χ2** | **P-value** | **Included in model?** |  |
| **Year** | **23** | **0.0029** | **Yes, as random effect** |  |
| **Altitude** | **2.9** | **0.087** | **Yes** |  |
| Altitude^2^ | 0.86 | 0.36 | Yes |  |
| Lay date | 0.58 | 0.44 | Yes |  |
| Lay date^2^ | 0.16 | 0.69 | Yes |  |
| Hatch date | 0.73 | 0.39 | No, correlation with lay date |  |
| Hatch date^2^ | 0.015 | 0.90 | No, correlation with lay date |  |
| **Clutch size** | **5.6** | **0.018** | **No, correlation with brood size** |  |
| Clutch size^2^ | 0.58 | 0.45 | No, correlation with brood size |  |
| **Brood size** | **8.50** | **0.0036** | **Yes** |  |
| Brood size^2^ | 0.057 | 0.81 | Yes |  |
| Mean temp. in-nest | 0.019 | 0.89 | Yes |  |
| Mean temp. in-nest^2^ | 0.21 | 0.65 | Yes |  |
| Total rainfall in-nest | 0.49 | 0.48 | Yes |  |
| Total rainfall in-nest^2^ | 0.39 | 0.53 | Yes |  |

| Table S8. Single term (and two term) model outputs for variables influencing probability of at least one fledgling being recruited into the local population from the full dataset but including only nests which fledged. Used to determine terms for subsequent inclusion in the Binomially distributed GLMM ‘Local recruitment all years’. Terms with P ≤ 0.1 in bold. N = 197. | | | |
| --- | --- | --- | --- |
| **Variable** | **χ2** | **P-value** | **Included in model?** |
| Year | 9.2 | 0.32 | Yes, as random effect |
| Altitude | 0.83 | 0.36 | Yes |
| Altitude^2^ | 1.9 | 0.17 | Yes |
| **Lay date** | **9.5** | **0.0021** | **No, correlation with hatch date** |
| Lay date^2^ | 1.9 | 0.17 | No, correlation with hatch date |
| **Hatch date** | **8.7** | **0.0032** | **Yes** |
| Hatch date^2^ | 2.0 | 0.16 | Yes |
| **Clutch size** | **8.3** | **0.0039** | **No, correlation with brood size** |
| Clutch size^2^ | 0.030 | 0.86 | No, correlation with brood size |
| **Brood size** | **9.5** | **0.0021** | **Yes** |
| Brood size^2^ | <0.001 | 0.98 | Yes |
| **Mean temp. in-nest** | **6.9** | **0.0087** | **Yes** |
| Mean temp. in-nest^2^ | 1.4 | 0.23 | Yes |
| Total rainfall in-nest | 2.0 | 0.16 | Yes |
| Total rainfall in-nest^2^ | 1.1 | 0.29 | Yes |
| Mean temp. 50d post-fledge | 0.25 | 0.62 | No, correlation with temp. 40d |
| Mean temp. 50d post-fledge^2^ | 2.5 | 0.12 | No, correlation with temp. 40d |
| Mean temp. 40d post-fledge | 0.13 | 0.72 | Yes |
| Mean temp. 40d post-fledge^2^ | 2.6 | 0.11 | Yes |
| Total rainfall 50d post-fledge | 0.55 | 0.46 | No, correlation with rainfall 40d |
| Total rainfall 50d post-fledge^2^ | 0.52 | 0.47 | No, correlation with rainfall 40d |
| Total rainfall 40d post-fledge | 0.14 | 0.70 | Yes |
| Total rainfall 40d post-fledge^2^ | 1.6 | 0.21 | Yes |

| Table S9. Single term (and two term) model outputs for variables influencing the proportion of fledglings which were recruited into the local population from the full dataset but including only nests which fledged. Used to determine terms for subsequent inclusion in the Binomially distributed GLMM ‘Proportion recruited all years’. Terms with P ≤ 0.1 in bold. N = 197. | | | |
| --- | --- | --- | --- |
| **Variable** | **χ2** | **P-value** | **Included in model?** |
| **Year** | **15** | **0.065** | **Yes, as random effect** |
| Altitude | 0.91 | 0.34 | Yes |
| Altitude^2^ | 0.19 | 0.67 | Yes |
| Lay date | 2.2 | 0.14 | Yes |
| **Lay date^2^** | **3.4** | **0.066** | **Yes** |
| Hatch date | 2.1 | 0.15 | No, correlation with lay date |
| Hatch date^2^ | 2.6 | 0.11 | No, correlation with lay date |
| Clutch size | 0.90 | 0.34 | Yes |
| Clutch size^2^ | 0.24 | 0.63 | Yes |
| Brood size | 1.1 | 0.29 | No, correlation with clutch size |
| Brood size^2^ | 0.096 | 0.76 | No, correlation with clutch size |
| Mean temp. in-nest | 0.17 | 0.68 | Yes |
| Mean temp. in-nest^2^ | 1.9 | 0.17 | Yes |
| **Total rainfall in-nest** | **3.6** | **0.058** | **Yes** |
| **Total rainfall in-nest^2^** | **2.8** | **0.095** | **Yes** |
| Mean temp. 50d post-fledge | 0.21 | 0.64 | No, correlation with temp 40d |
| **Mean temp. 50d post-fledge^2^** | **4.7** | **0.030** | **No, correlation with temp 40d** |
| Mean temp. 40d post-fledge | 0.19 | 0.67 | Yes |
| **Mean temp. 40d post-fledge^2^** | **8.1** | **0.0045** | **Yes** |
| Total rainfall 50d post-fledge | 2.0 | 0.16 | No, correlation with rain 40d |
| Total rainfall 50d post-fledge^2^ | 0.6 | 0.43 | No, correlation with rain 40d |
| Total rainfall 40d post-fledge | 1.6 | 0.21 | Yes |
| **Total rainfall 40d post-fledge^2^** | **3.4** | **0.065** | **Yes** |

| Table S10. Spearman rank correlation coefficient matrix of all continuous and integer variables from the full dataset, including nests which fledged and nests which did not fledge. Utilised for analysis of the ‘Nest success full sample’ model. \|r_s_\| > 0.7 in bold. N = 247. | | | | | | | | |
| --- | --- | --- | --- | --- | --- | --- | --- | --- |
|  | Altitude | Lay date | Hatch date | Clutch size | Brood size | Mean temp. in-nest | Total rainfall in-nest |  |
| Total rainfall in-nest | .08 | .15 | .15 | -.08 | -.10 | -.21 |  |  |
| Mean temp. in-nest | .08 | .26 | .25 | -.19 | -.21 |  |  |  |
| Brood size | -.19 | -.37 | -.31 | **.82** |  |  |  |  |
| Clutch size | -.24 | -.41 | -.35 |  |  |  |  |  |
| Hatch date | .05 | **.96** |  |  |  |  |  |  |
| Lay date | .07 |  |  |  |  |  |  |  |
| Altitude |  |  |  |  |  |  |  |  |

| Table S11. Spearman rank correlation coefficient matrix of all continuous and integer variables from resighting data, including all nests which fledged. Utilised for analysis of models; ‘Local recruitment all years’ & ‘Proportion recruited all years’. \|r_s_\| > 0.7 in bold. N = 197. | | | | | | | | | | | |
| --- | --- | --- | --- | --- | --- | --- | --- | --- | --- | --- | --- |
|  | Altitude | Lay date | Hatch date | Clutch size | Brood size | Total rainfall 40d post-fledge | Total rainfall 50d post-fledge | Mean temp. 40d post-fledge | Mean temp. 50d post-fledge | Mean temp. in-nest | Total rainfall in-nest |
| Total rainfall in-nest | .11 | .13 | .13 | -.09 | -.12 | .06 | .00 | -.31 | -.24 | -.21 |  |
| Mean temp. in-nest | .02 | .27 | .24 | -.20 | -.23 | .26 | .08 | -.35 | -.43 |  |  |
| Mean temp. 50d post-fledge | -.15 | -.12 | -.04 | .14 | .19 | -.20 | .08 | **.96** |  |  |  |
| Mean temp. 40d post-fledge | -.16 | .01 | .08 | .08 | .15 | -.10 | .19 |  |  |  |  |
| Total rainfall 50d post-fledge | .08 | .13 | .14 | .10 | .11 | **.73** |  |  |  |  |  |
| Total rainfall 40d post-fledge | .07 | .29 | .27 | .04 | .00 |  |  |  |  |  |  |
| Brood size | -.19 | -.40 | -.33 | **.84** |  |  |  |  |  |  |  |
| Clutch size | -.23 | -.45 | -.37 |  |  |  |  |  |  |  |  |
| Hatch date | -.01 | **.98** |  |  |  |  |  |  |  |  |  |
| Lay date | .03 |  |  |  |  |  |  |  |  |  |  |
| Altitude |  |  |  |  |  |  |  |  |  |  |  |

| Table S12. Model outputs from the Binomially distributed GLMM ‘Nest success vegetation years’. Response variable – Nest success. Full model terms selected using single term analysis (Table S2) and removed by stepwise elimination until minimum adequate model (MAM) remained. Significant terms (P ≤ 0.05) in bold. N = 143. | | | | | |
| --- | --- | --- | --- | --- | --- |
| **Variable** | **Estimate ± SE** | **df** | **χ2** | **P-value** |  |
| (Intercept) | 2.19 ± 0.34 |  |  |  |  |
| **Brood size** | **0.92 ± 0.26** | **1, 143** | **12.37** | **<0.001** |  |
| **Bracken 50m** | **2.28 ± 0.93** | **1, 143** | **6.06** | **0.014** |  |
| **(Bracken 50m)^2^** | **-2.05 ± 0.95** | **1, 143** | **4.61** | **0.032** |  |
| Tree scrub 5m | -0.56 ± 0.32 | 1, 143 | 0.05 | 0.830 |  |
| Tree scrub 200m | -0.48 ± 0.25 | 1, 143 | 3.64 | 0.056 |  |
| Heather 50m | -0.17 ± 0.24 | 1, 143 | 0.48 | 0.488 |  |
| (Heather 50m)^2^ | -0.76 ± 0.77 | 1, 143 | 0.95 | 0.329 |  |
| Ditch/stream 5m | Y: -0.53 ± 1.36 | 1, 143 | 0.15 | 0.696 |  |
| Overhead wires 200m | Y: -0.19 ± 1.34 | 1, 143 | 0.02 | 0.887 |  |
| Path 50m | Y: -0.85 ± 0.56 | 1, 143 | 2.26 | 0.133 |  |
| **Mean temp. in-nest** | **-11.37 ± 4.84** | **1, 143** | **5.51** | **0.019** |  |
| **Mean temp. in-nest^2^** | **11.03 ± 4.70** | **1, 143** | **5.52** | **0.019** |  |

| Table S13. Model outputs from the Binomially distributed GLMM ‘Nest success all years‘. Response variable – Nest success. Full model terms selected using single term analysis (Table S7) and removed by stepwise elimination until minimum adequate model (MAM) remained. Significant terms (P ≤ 0.05) in bold. N = 247. | | | | | |
| --- | --- | --- | --- | --- | --- |
| **Variable** | **Estimate ± SE** | **df** | **χ2** | **P-value** |  |
| (Intercept) | 1.44 ± 0.28 |  |  |  |  |
| **Altitude** | **0.42 ± 0.19** | **1, 247** | **5.06** | **0.024** |  |
| Altitude^2^ | -2.31 ± 2.68 | 1, 247 | 1.03 | 0.309 |  |
| Lay date | -0.03 ± 0.22 | 1, 247 | 0.01 | 0.907 |  |
| Lay date^2^ | 0.29 ± 1.19 | 1, 247 | 0.06 | 0.805 |  |
| **Brood size** | **0.56 ± 0.17** | **1, 247** | **10.63** | **0.001** |  |
| Brood size^2^ | 0.02 ± 0.93 | 1, 247 | 0.00 | 0.983 |  |
| Mean temp. in-nest | 0.16 ± 0.20 | 1, 247 | 0.68 | 0.408 |  |
| Mean temp. in-nest^2^ | 2.22 ± 2.73 | 1, 247 | 0.66 | 0.417 |  |
| Total rainfall in-nest | 0.18 ± 0.19 | 1, 247 | 0.85 | 0.356 |  |
| Total rainfall in-nest^2^ | -0.36 ± 0.61 | 1, 247 | 0.34 | 0.560 |  |

| Table S14. Model outputs from the Poisson distributed GLMM ‘Local recruitment vegetation years‘. Response variable – Local recruitment. Full model terms selected using single term analysis (Table S3) and removed by stepwise elimination until minimum adequate model (MAM) remained. Significant terms (P ≤ 0.05) in bold. N = 118. | | | | | |
| --- | --- | --- | --- | --- | --- |
| **Variable** | **Estimate ± SE** | **df** | **χ2** | **P-value** |  |
| (Intercept) | 0.76 ± 0.21 |  |  |  |  |
| Altitude | 0.36 ± 0.23 | 1, 118 | 2.52 | 0.11 |  |
| Altitude^2^ | -2.56 ± 2.78 | 1, 118 | 0.65 | 0.419 |  |
| Lay date | -0.19 ± 0.26 | 1, 118 | 0.53 | 0.469 |  |
| **Brood size** | **0.73 ± 0.22** | **1, 118** | **11.20** | **<0.001** |  |
| Bracken 200m | -0.06 ± 0.32 | 1, 118 | 0.04 | 0.844 |  |
| Rush 50m | -0.11 ± 0.29 | 1, 118 | 0.15 | 0.703 |  |
| Rush 200m | -0.26 ± 0.22 | 1, 118 | 1.35 | 0.245 |  |
| Bilberry 5m | -0.38 ± 0.21 | 1, 118 | 4.68 | 0.063 |  |
| Heather 50m | 0.03 ± 0.26 | 1, 118 | 0.01 | 0.924 |  |
| **Heather 200m** | **-0.50 ± 0.20** | **1, 118** | **6.12** | **0.013** |  |
| Path 5m | Y: 1.50 ± 0.79 | 1, 118 | 3.61 | 0.057 |  |
| Mean temp. in-nest | -0.02 ± 0.37 | 1, 118 | 0.00 | 0.959 |  |
| Mean temp. 50d post-fledge | 0.31 ± 0.22 | 1, 118 | 2.06 | 0.151 |  |

| Table S15. Model outputs from the Binomially distributed GLMM ‘Local recruitment all years‘. Response variable – Local recruitment. Full model terms selected using single term analysis (Table S8) and removed by stepwise elimination until minimum adequate model (MAM) remained. Significant terms (P ≤ 0.05) in bold. N = 197. | | | | | |
| --- | --- | --- | --- | --- | --- |
| **Variable** | **Estimate ± SE** | **df** | **χ2** | **P-value** |  |
| (Intercept) | 0.82 ± 0.50 |  |  |  |  |
| Altitude | 0.24 ± 0.17 | 1, 197 | 1.96 | 0.162 |  |
| Altitude^2^ | 3.40 ± 1.94 | 1, 197 | 2.70 | 0.100 |  |
| Hatch date | -0.36 ± 0.19 | 1, 197 | 3.70 | 0.054 |  |
| Hatch date^2^ | -0.30 ± 2.15 | 1, 197 | 0.02 | 0.888 |  |
| **Brood size** | **0.50 ± 0.16** | **1, 197** | **9.45** | **0.002** |  |
| Brood size^2^ | -0.05 ± 1.00 | 1, 197 | 0.00 | 0.959 |  |
| Mean temp. in-nest | -0.17 ± 0.31 | 1, 197 | 0.32 | 0.572 |  |
| Mean temp. in-nest^2^ | 0.64 ± 3.77 | 1, 197 | 0.03 | 0.864 |  |
| Total rainfall in-nest | -0.22 ± 0.20 | 1, 197 | 0.05 | 0.829 |  |
| Total rainfall in-nest^2^ | -0.10 ± 0.61 | 1, 197 | 0.02 | 0.874 |  |
| Mean temp. 40d post-fledge | 0.02 ± 0.25 | 1, 197 | 0.01 | 0.932 |  |
| Mean temp. 40d post-fledge^2^ | 7.33 + 6.91 | 1, 197 | 1.12 | 0.289 |  |
| Total rainfall 40d post-fledge | 0.10 ± 0.20 | 1, 197 | 0.23 | 0.635 |  |
| Total rainfall 40d post-fledge^2^ | -1.03 ± 0.75 | 1, 197 | 1.91 | 0.167 |  |

| Table S16. Model outputs from the Poisson distributed GLMM ‘Proportion recruited vegetation years‘. Response variable – Proportion recruited. Full model terms selected using single term analysis (Table S4) and removed by stepwise elimination until minimum adequate model (MAM) remained. Significant terms (P ≤ 0.05) in bold. N = 118. | | | | | |
| --- | --- | --- | --- | --- | --- |
| **Variable** | **Estimate ± SE** | **df** | **χ2** | **P-value** |  |
| (Intercept) | -1.40 ± 0.19 |  |  |  |  |
| **Brood size** | **0.42 ± 0.15** | **1, 118** | **7.63** | **0.006** |  |
| **Tree scrub 200m** | **0.88 ± 0.36** | **1, 118** | **5.97** | **0.015** |  |
| **(Tree scrub 200m)^2^** | **-0.90 ± 0.39** | **1, 118** | **5.32** | **0.021** |  |
| Other grass 200m | 0.09 ± 0.10 | 1, 118 | 0.83 | 0.364 |  |
| Rush 200m | -0.16 ± 0.12 | 1, 118 | 1.94 | 0.163 |  |
| **Heather 50m** | **-0.88 ± 0.32** | **1, 118** | **7.68** | **0.006** |  |
| **(Heather 50m)^2^** | **0.83 ± 0.31** | **1, 118** | **7.30** | **0.007** |  |
| Fence posts 5m | Y: 0.59 ± 0.48 | 1, 118 | 1.32 | 0.251 |  |
| Path 200m | Y: 0.32 ± 0.27 | 1, 118 | 1.43 | 0.233 |  |
| **Total rainfall in-nest** | **-0.38 ± 0.14** | **1, 118** | **7.02** | **0.008** |  |
| Total rainfall in-nest^2^ | -0.82 ± 0.47 | 1, 118 | 3.10 | 0.078 |  |
| Mean temp. 50d post-fledge | 0.07 ± 0.11 | 1, 118 | 0.42 | 0.519 |  |

| Table S17. Model outputs from the Binomially distributed GLMM ‘Proportion recruited all years‘. Response variable – Proportion recruited. Full model terms selected using single term analysis (Table S9) and removed by stepwise elimination until minimum adequate model (MAM) remained. Significant terms (P ≤ 0.05) in bold. N = 197. | | | | | |
| --- | --- | --- | --- | --- | --- |
| **Variable** | **Estimate ± SE** | **df** | **χ2** | **P-value** |  |
| (Intercept) | -1.14 ± 0.09 |  |  |  |  |
| Altitude | 0.05 ± 0.08 | 1, 197 | 0.34 | 0.560 |  |
| Altitude^2^ | -0.82 ± 0.96 | 1, 197 | 0.72 | 0.397 |  |
| Lay date | -0.13 ± 0.10 | 1, 197 | 1.86 | 0.173 |  |
| Lay date^2^ | 0.02 ± 0.70 | 1, 197 | 0.00 | 0.982 |  |
| Clutch size | 0.00 ± 0.10 | 1, 197 | 0.00 | 0.979 |  |
| Clutch size^2^ | 0.17 ± 0.79 | 1, 197 | 0.05 | 0.829 |  |
| Mean temp. in-nest | -0.08 ± 0.13 | 1, 197 | 0.39 | 0.531 |  |
| Mean temp. in-nest^2^ | 1.26 ± 1.37 | 1, 197 | 0.84 | 0.359 |  |
| Total rainfall in-nest | -1.16 ± 0.10 | 1, 197 | 3.11 | 0.078 |  |
| Total rainfall in-nest^2^ | -0.57 ± 0.37 | 1, 197 | 0.80 | 0.372 |  |
| **Mean temp. 40d post-fledge** | **-7.70 ± 2.71** | **1, 197** | **8.07** | **0.004** |  |
| **Mean temp 40d post-fledge^2^** | **7.69 ± 2.71** | **1, 197** | **8.05** | **0.005** |  |
| Total rainfall 40d post-fledge | 0.04 ± 0.09 | 1, 197 | 0.17 | 0.683 |  |
| Total rainfall 40d post-fledge^2^ | -0.60 ± 0.37 | 1, 197 | 2.61 | 0.106 |  |

**Mayfield survival analysis**

From 2015-2021 detailed records were kept on the date that each nest was found, the initial condition and the last recorded date of nest activity. This allowed us to perform a nest survival analysis on this subset of our data to estimate nest survival probability more accurately. First, we recorded the day of first activity, this was either the lay date if a nest was found prior to laying or the day of first discovery. Secondly, we estimated the day of last activity which was either the fledge date (successful nests) or the intermediate date between the last day of recorded nest activity and first day of recorded inactivity. The time between these dates was considered the total number of days a nest was exposed to predation risk. The daily nest survival was then estimated from the following equation.

$$Daily nest survival probability=\frac{Total exposure days-Total number of failed nests}{Total exposure days}$$

To estimate overall nest survival probability, we then calculated the average number of days from first egg laying until successful fledgling, which we applied in the following equation.

$$Nest survival probability={Daily nest survival probability}^{Average nesting duration}$$

**Brood size analysis**

**
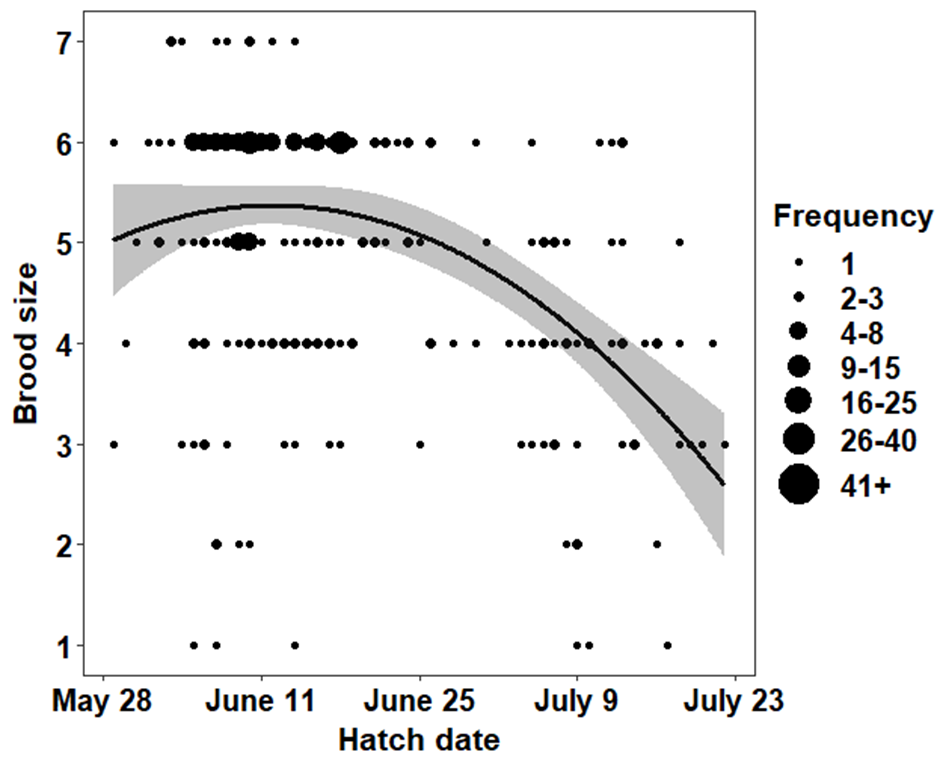
**

Fig. S3. In-nest brood size declines with later hatch date, from all years (N = 247 nests). To visualise overlapping data points the size of each point depends on frequency of occurrence of nests with the same hatch date and brood size. Predicted relationships (± 95% CI) are fitted from GLMMs, see Table S18.

| Table S18. GLMM outputs for variables influencing brood size. Analysis performed on data from years with vegetation and landmark features sampled (143 out of 247 broods) and from all years (N = 247). | | | | |
| --- | --- | --- | --- | --- |
| **Fixed effects** | **Estimates ± SE** | **df** | **χ2** | **P-value** |
| 1. **Brood size vegetation years** | | | | |
| Hatch date | 0.38 ± 0.18 | 1, 143 | 4.39 | 0.036 |
| Hatch date^2^ | -0.51 ± 0.19 | 1, 143 | 7.50 | 0.007 |
|  |  |  |  |  |
| 1. **Brood size all years** | | | | |
| Hatch date | 0.46 ± 0.16 | 1, 247 | 7.65 | 0.006 |
| Hatch date^2^ | -0.58 ± 0.17 | 1, 247 | 12.07 | <0.001 |

To establish whether brood size varied throughout the breeding season in the anticipated manner within our samples we ran a Conway-Maxwell Poisson distributed generalised linear mixed effects model using the package glmmTMB (Brooks et al. 2017) for the vegetation sample (N = 143) and the full sample (N = 247). The response variable for these models was the brood size, the fixed effects were the number of days since March 1 of each year that a brood hatched. Year was also included as a random effect (Table S18, Fig. S3).

**Principle components analysis**

| Table S19. Relevant PCA statistics for all relative vegetation cover variables sampled at N = 143 | | | | | | | | |
| --- | --- | --- | --- | --- | --- | --- | --- | --- |
|  | PC1 | PC2 | PC3 | PC4 | PC5 | PC6 | PC7 | PC8 |
| Standard deviation | 1.437 | 1.184 | 1.065 | 1.014 | 0.938 | 0.886 | 0.838 | 0.031 |
| Proportion of variance | 0.258 | 0.175 | 0.142 | 0.129 | 0.110 | 0.098 | 0.089 | <0.000 |
| Cumulative proportion | 0.258 | 0.433 | 0.575 | 0.704 | 0.814 | 0.912 | 1.000 | 1.000 |

| Table S20. PCA loading variables for PCs containing all relative vegetation cover variables sampled at N = 143 Whinchat nests. | | | | | | | | |
| --- | --- | --- | --- | --- | --- | --- | --- | --- |
|  | PC1 | PC2 | PC3 | PC4 | PC5 | PC6 | PC7 | PC8 |
| Bracken | 0.625 | -0.294 | 0.134 | 0.169 | -0.078 | 0.147 | 0.003 | 0.670 |
| Tree scrub | -0.337 | -0.118 | 0.524 | 0.087 | 0.058 | 0.401 | -0.650 | 0.057 |
| Tufted hair grass | -0.376 | -0.290 | 0.135 | -0.190 | -0.273 | 0.498 | 0.623 | 0.101 |
| Purple moor grass | 0.005 | 0.604 | 0.245 | -0.481 | 0.428 | 0.086 | 0.144 | 0.363 |
| Other grass | -0.438 | -0.338 | -0.467 | -0.299 | 0.118 | -0.259 | -0.234 | 0.501 |
| Rush | -0.360 | 0.159 | 0.406 | 0.461 | -0.163 | -0.544 | 0.224 | 0.308 |
| Bilberry | -0.175 | 0.182 | -0.395 | 0.628 | 0.449 | 0.393 | 0.117 | 0.129 |
| Heather | -0.050 | 0.527 | -0.301 | 0.014 | -0.701 | 0.210 | -0.224 | 0.208 |


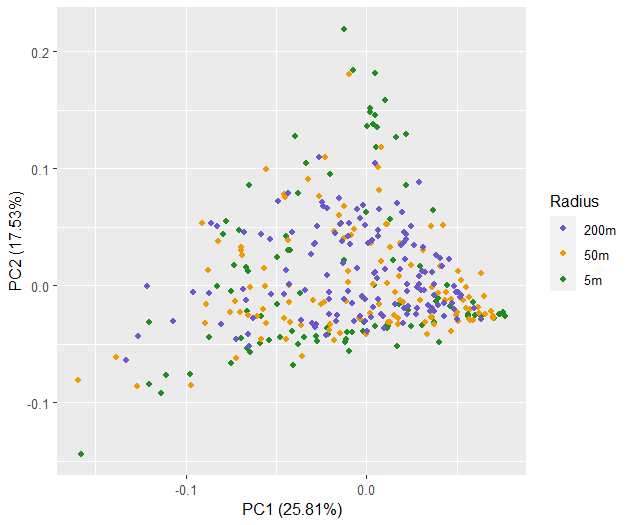


Figure S4. Scatterplot of PC1 vs PC2 generated from PCA of all relative vegetation cover variables sampled at N = 143 Whinchat nests. Colours represent radius within which vegetation was sampled (Blue = 200m, Orange = 50m & Green = 5m).

References

Mollie E. Brooks, Kasper Kristensen, Koen J. van Benthem, Arni Magnusson, Casper W. Berg, Anders Nielsen, Hans J. Skaug, Martin Maechler and Benjamin M. Bolker (2017). glmmTMB Balances Speed and Flexibility Among Packages for Zero-inflated Generalized Linear Mixed Modeling. The R Journal, 9(2), 378-400. doi: 10.32614/RJ-2017-066.
